# GNAT toxins evolve toward narrow tRNA target specificities

**DOI:** 10.1093/nar/gkac356

**Published:** 2022-05-24

**Authors:** Dmitry Bikmetov, Alexander M J Hall, Alexei Livenskyi, Bridget Gollan, Stepan Ovchinnikov, Konstantin Gilep, Jenny Y Kim, Gerald Larrouy-Maumus, Viktor Zgoda, Sergei Borukhov, Konstantin Severinov, Sophie Helaine, Svetlana Dubiley

**Affiliations:** Center for Precision Genome Editing and Genetic Technologies for Biomedicine, Institute of Gene Biology, Russian Academy of Sciences, Moscow 119334, Russia; Institute of Molecular Genetics of National Research Center «Kurchatov Institute», Moscow 123182, Russia; Department of Microbiology, Harvard Medical School, Boston, MA 02115, USA; Center for Precision Genome Editing and Genetic Technologies for Biomedicine, Institute of Gene Biology, Russian Academy of Sciences, Moscow 119334, Russia; Faculty of Bioengineering and Bioinformatics, Lomonosov Moscow State University, Moscow 119992, Russia; Department of Microbiology, Harvard Medical School, Boston, MA 02115, USA; Center for Life Sciences, Skolkovo Institute of Science and Technology, Skolkovo 143025, Russia; Center for Precision Genome Editing and Genetic Technologies for Biomedicine, Institute of Gene Biology, Russian Academy of Sciences, Moscow 119334, Russia; Department of Microbiology, Harvard Medical School, Boston, MA 02115, USA; MRC Centre for Molecular Bacteriology and Infection, Imperial College London, London SW7 2AZ, UK; Institute of Biomedical Chemistry, Moscow 119435, Russia; Department of Cell Biology and Neuroscience, Rowan University School of Osteopathic Medicine, Stratford, NJ 08084-1489, USA; Institute of Molecular Genetics of National Research Center «Kurchatov Institute», Moscow 123182, Russia; Center for Life Sciences, Skolkovo Institute of Science and Technology, Skolkovo 143025, Russia; Department of Microbiology, Harvard Medical School, Boston, MA 02115, USA; Center for Life Sciences, Skolkovo Institute of Science and Technology, Skolkovo 143025, Russia; Institute of Gene Biology, Russian Academy of Science, 119334 Moscow, Russia

## Abstract

Type II toxin–antitoxin (TA) systems are two-gene modules widely distributed among prokaryotes. GNAT toxins associated with the DUF1778 antitoxins represent a large family of type II TAs. GNAT toxins inhibit cell growth by disrupting translation via acetylation of aminoacyl-tRNAs. In this work, we explored the evolutionary trajectory of GNAT toxins. Using LC/MS detection of acetylated aminoacyl-tRNAs combined with ribosome profiling, we systematically investigated the *in vivo* substrate specificity of an array of diverse GNAT toxins. Our functional data show that the majority of GNAT toxins are specific to Gly-tRNA isoacceptors. However, the phylogenetic analysis shows that the ancestor of GNAT toxins was likely a relaxed specificity enzyme capable of acetylating multiple elongator tRNAs. Together, our data provide a remarkable snapshot of the evolution of substrate specificity.

## INTRODUCTION

Toxin-antitoxin (TA) systems are a large and diverse group of gene loci present in various organisms across the bacterial and archaeal domains of life (1). TA systems comprise two components: a non-secreted toxin that inhibits an essential cellular process, such as mRNA translation, DNA replication, or membrane homeostasis (2), and an antitoxin that blocks the action of the toxin. While some TA systems have been implicated in the bacterial response to specific stresses, including phage infection (3,4) or attacks by host innate immunity (5,6), the biological function of most remains unknown.

There are many mechanisms by which toxins corrupt their targets, including phosphorylation (7), AMPylation (8), ADP-ribosylation (9), inhibitory protein–protein interactions (10), and nucleic acids degradation (11). TA systems are currently classified into eight major types defined by the mode of antitoxin action (12), with type II TA systems being the most diverse and extensively studied. Type II antitoxins are proteins that inhibit their cognate toxin through direct protein–protein interactions. The antitoxins are typically encoded in a single operon with the toxin.

The Gcn-5-like *N*-acetyltransferase (GNAT) toxins are components of the numerous type II TA (13–19) systems, of which only a handful have been characterized. These toxins acetylate the α-amino group of amino acids on charged (aminoacylated) tRNA (13), making it unusable by the ribosome. Upon overexpression, GNAT toxins inhibit translation and arrest cell growth, presumably through depletion of charged tRNAs needed for protein synthesis. To date, targets of six GNAT toxins have been characterized *in vitro* and/or *in vivo*. *In vitro*, the AtaT toxin from *Escherichia coli* O157:H7 was shown to exclusively acetylate initiator Met-tRNA^fMet^ (17). However, *in vivo*, AtaT overproduced from a plasmid exhibited a broad specificity toward different elongator tRNAs (20). The ItaT toxin displayed a narrow specificity toward Ile-tRNA^Ile^ *in vitro* (15), whereas *in vivo*, overexpression of ItaT resulted in acetylation of a number of tRNAs (21). Conversely, while the initial *in vitro* study of TacT, TacT2, and TacT3 toxins from *Salmonella enterica* serovar Typhimurium revealed a relaxed specificity toward several elongator tRNAs (14), *in vivo* TacT was shown to be specific to Gly-tRNA^Gly^ only (22). AtaT2, another GNAT toxin encoded by *E. coli* O157:H7, was shown to arrest ribosomes at glycine codons *in vivo* and *in vitro* (16). Thus, the understanding of the specificity and physiological targets of GNAT toxins remains incomplete.

In this work, we investigated *in vivo* substrate specificity of fourteen diverse GNAT toxins from different bacterial species. Eight toxins exclusively acetylated Gly-tRNA^Gly^, while the rest displayed broader substrate specificity. Our comprehensive phylogenetic analysis of all GNAT toxins present in the sequenced bacterial genome database reveals that the narrow-specificity GNAT toxins are likely descendants of the evolutionarily ancient, promiscuous tRNA acetyltransferases. We suggest that evolutionary pressures have led to the specialization of GNAT toxins towards a limited range of target elongator aa-tRNAs.

## MATERIALS AND METHODS

### Plasmid сonstruction

Plasmids, primers, and bacterial strains used in this project are listed in Supplementary Tables S1–S3.

Genes of interest were cloned into target vectors by standard restriction cloning procedures using Q5 polymerase, restriction endonucleases, and T4 ligase reagents from New England Biolabs. Genes of *ataT*, *gmvT*, *kacT*, *tacT_Bcn_*, *vcaT, acaT, vcaAT, sonT, itaT*,*ataT2*, and *tacT_Ret_* were PCR amplified from synthetic DNA fragments purchased from GeneArt Gene Synthesis Services (ThermoFisher), Quintara Biosciences, and Integrated DNA Technologies. GNAT toxins were cloned into pBAD30 and pBAD33 vectors (23) using SalI and HindIII restriction sites to generate arabinose-inducible overexpression vectors. All constructed plasmids were verified by Sanger sequencing (Qintara Biosciences). The GNAT toxin sequences and accession numbers for their source are available in Supplementary Table S4.

### Cell toxicity tests

Bacterial strains were sequentially transformed with the pCA24N-*pth* (24) and pBAD30-GNAT toxin overexpression vectors or the appropriate empty vectors to act as controls. TacT3 was expressed in a *Salmonella enterica* subspecies *enterica* serovar Typhimurium str. 14028 background strain, whereas AtaT and ItaT were expressed in *Escherichia coli* DH5α. Stationary phase bacterial cultures were inoculated to an OD_600_ of 0.01 in fresh LB media containing 50 μg/ml carbenicillin and 34 μg/ml chloramphenicol. Media was also supplemented with 0.5% w/v l-arabinose and 0.1 mM IPTG (for TacT3 and AtaT-expressing strains) or 0.02% (w/v) l-arabinose and 0.4 mM IPTG (for ItaT-expressing strains). Five milliliter cultures were incubated at 37°C with orbital shaking, and OD_600_ measurements were taken hourly. Three biological replicates were prepared for each strain, and the plotted data represent the arithmetic mean of the replicates, with standard deviation shown by the error bars.

A DNA fragment encoding the 3xFLAG tag was inserted into the pBAD33 plasmid cut with HindIII restriction nuclease to create the pBAD33-3xFLAG plasmid. DNA fragments coding for VcaT and VcaAT were inserted into the pBAD33-3xFLAG vector between SacI and SalI sites to allow expression of VcaT C-terminally fused with the 3xFLAG sequence. *E. coli* BW25113 cells were transformed with pBAD33-*vcaT-FLAG3*, pBAD33-*vcaAT-FLAG3* and an empty vector, which was used as a negative control. Cells were grown overnight on 2× YT agar plates supplemented with 1.0% glucose and 34 μg/ml chloramphenicol. A single colony from the freshly transformed plate was inoculated into 10 ml of 2× YT medium containing 34 μg/ml chloramphenicol and grown to OD_600_ ∼0.3 at 37°C. Cell cultures were divided into two aliquots and grown at 37°C in the presence or absence of 20 mM arabinose for additional 60 min. Serial 10-fold dilutions of the cultures were plated on 2× YT agar supplemented with 34 μg/ml chloramphenicol and 1.0% glucose. Cells from the remaining 5 ml of induced cultures were pelleted, resuspended in 250 μl of the BW buffer (50 mM Tris–HCl, pH 8.0, 300 mM NaCl) supplemented with 3mM β-mercaptoethanol and 1 mM phenylmethanesulfonyl fluoride. Cells were disrupted by sonication and cell debris was removed by centrifugation at 21 000 × *g* at 4°C. The total protein concentrations in lysates was measured by the Bradford assay (Protein Assay Reagent, Bio-Rad, USA) according to the manufacturer's protocol. Samples were loaded on the Laemmli 13% SDS-PAAG (the set of samples was loaded twice on different sides of the gel). After electrophoresis, the gel was cut in halves. One half was stained with EZblue protein stain (Sigma-Aldrich, USA) according to the manufacturer's protocol. The other part of the gel was used for the Western blotting. The Western blot was developed with mouse monoclonal ANTI-FLAG^®^ M2 antibody (F3165, Merck, USA) and Anti-Mouse IgG (whole molecule)–Peroxidase rabbit antibody (A9044, Merck, USA). The membrane was developed using Clarity™ Western ECL Substrate (Bio-Rad Laboratories, USA) according to the manufacturer's protocol. The luminescence was recorded using Vilber Fusion FX gel documentation system (Vilber Lourmat, France) and the manufacturer's software.

### Analysis of the ratio of *in vivo* acetylated of tRNA isoacceptors

A single colony of *E. coli* BW25113 transformed with pBAD33, pBAD33-*ataT*, pBAD33-*ataT2*, pBAD33-*itaT* or pBAD-*tacT3* was inoculated into 2× YT medium supplemented with 34 μg/ml chloramphenicol and 0.25% glucose. Cells were grown at 37°C for 3 h. One milliliter of the culture was collected, cells were washed with a fresh medium to remove glucose, transferred into a 50 ml 2× YT medium containing 34 μg/ml chloramphenicol, and allowed to grow to OD_600_ ∼0.3. Toxin expression was induced with 1 mM arabinose. Ten milliliter aliquots of cultures were collected by centrifugation at 5- and 45-min post-induction time points. For purification of tRNA-enriched fraction, cell pellets were resuspended in 0.5 ml of 300 mM Na-acetate buffer (pH 4.5)/10 mM EDTA and mixed with 0.5 ml of phenol saturated with 0.1 M citrate buffer (pH 4.3). Extraction was carried out at 4°C with gentle rotation for 15 min. After phase separation, the aqueous phase was collected, and the phenol extraction step was repeated. The resulting RNA was precipitated with 1 ml of isopropanol; the precipitate was washed with 100 mM Na-acetate buffer (pH 4.5) in 70% ethanol and air-dried. To prepare tRNA samples from toxin-expressing cells for LC/MS, 50 μg of purified tRNA was dissolved in 80% DMSO containing 5 mM Fmoc *N*-hydroxysuccinimide ester (Fmoc-OSu). The reaction mixture was incubated at 4°C for 16 h and quenched by ethanol precipitation. To calculate the Relative Response Factor (RRF) of Fmoc- and acetyl derivatives of individual aminoacyl-adenosine molecules, tRNA purified from *E. coli* BW25113 harboring a pBAD33 empty vector was divided into two equal aliquots. One aliquot was treated with Fmoc-OSu as described above, and the second aliquot was used for acetylation. For acetylation, 50 μg of tRNA was dissolved in 250 μl of 200 mM Na-acetate buffer (pH 5.2), followed by the addition of 4 μl of acetic anhydride. The reaction was incubated on ice for 1 h, then supplemented with an additional 4 μl of acetic anhydride, followed by 1-h incubation on ice. Upon reaction completion, the concentration of Na-acetate was adjusted to 300 mM, and the acetylated tRNA was precipitated by the addition of 3 volumes of ethanol. Thirty microgams of aminoacyl-tRNA modified with Fmoc or acetyl group was digested with 100 U of RNase I (Ambion, USA) and 1000 U of RNase T1 (Thermo Scientific, USA) in 25 mM ammonium acetate (pH 7.0) at 37°C for 30 min. After quantification, the RRF for each aminoacyl-adenosine type was calculated as the ratio of [M + H]^+^ peak areas of the corresponding Fmoc- and acetyl derivatives.

LC/MS analysis was performed using Agilent 1200 HPLC equipped with the UV and 6550 iFunnel QTOF LC/MS detectors and Jet Stream Technology ion source (Agilent, USA). The products of RNase I digestion were separated by Poroshell 120 SB-C18 column (2.7 μm, 2.1 × 100 mm) (Agilent, USA) at 40°C using a linear (0–80%) gradient of acetonitrile in 5 mM ammonium acetate buffer (pH 5.2) at 0.2 ml/min. The electrospray source was set to positive ion mode at 4 kV, 290°C. Data acquisition was performed in the *m/z* range 200–1100 at 2 spectra/s rate. Fragmentation spectra were recorded in AutoMSMS mode. Data were analyzed using MassHunter Qualitative Analysis B.05.00 software (Agilent, USA) with Quartic/Quintic Savitzky-Golay smoothing function (function width: 30 points) and ChemStation integrator selector (default options, but advanced baseline correction mode).

Deconvolution of overlapped leucine- and isoleucine-adenosine peaks was performed using an exponentially modified Gaussian model fitted with the Python package ‘LMFIT’ version 1.0.3 (DOI: 10.5281/zenodo.5570790). Standard deviations of the fractions of acetylated aminoacyl-tRNAs were calculated using Python ‘uncertainties’ package version 3.1.6 to account for the propagation of error (E.O. Lebigot, https://pythonhosted.org/uncertainties/). Python scripts used for LC/MS data analysis are available in the GitHub repository (https://github.com/bikdm12/GNAT_toxins).

### Analysis of patterns of *in vivo* acetylated tRNA

Bacterial strains were transformed with pBAD30 GNAT toxin overexpression vectors. TacT, TacT2, and TacT3 were expressed in a *Salmonella enterica* subspecies *enterica* serovar Typhimurium str. 14028 background strain, and AcaT, VcaT, SonT, AtaT, ItaT, GmvT, KacT, TacT_Bcn_, and TacT_Ret_ were expressed in *Escherichia coli* DH5α. Stationary phase cultures were diluted to an OD_600_ of 0.05 in 15 ml fresh LB media in a 50 ml conical tube. Cultures were supplemented with 50 μg/ml carbenicillin and incubated at 37°C with orbital shaking for 3 h, at which point the cultures were supplemented with 0.5% (w/v) l-arabinose and incubated for additional 3 h. Bacteria were pelleted by centrifugation (10 min, 4000 × *g*, 4°C), transferred to 1.5 ml microcentrifuge tubes, flash-frozen in liquid nitrogen, and stored at –80°C until RNA extraction. Whole-cell RNA was isolated using an acid-phenol extraction protocol with all steps carried out cooled on ice. Bacterial pellets were thawed and resuspended in 700 μl sodium acetate buffer (300 mM sodium acetate pH 5.2, 10 mM EDTA). Next, 700 μl phenol saturated with 0.1 M Na-citrate buffer pH 4.3 (Sigma P4682) was added to each sample, and suspensions were incubated on ice for 15 min with regular vortexing. The aqueous phase was separated by centrifugation (12 000 x *g*, 15 min, 4°C), and retained. An additional 300 μl Na-acetate buffer was added to the phenol, followed by an additional round of vortexing and centrifugation. The aqueous phase from the two rounds of extraction was pooled, RNA was precipitated by 2.5 volumes of ice-cold ethanol, and incubated at–20°C overnight. The RNA precipitate was collected by centrifugation (21 000 × *g*, 15 min at 4°C), resuspended in 10 mM ammonium acetate, and stored at –80°C until required for downstream analysis.

The purified RNA (15–50 μg) was spiked with 1 μM ^15^N-AMP and incubated with 1 U of Nuclease P1 in 10 mM ammonium acetate for 30 min at 25°C. Alternatively, purified RNA was incubated with 25 μg of purified Pth in 10 mM Tris-acetate buffer, pH 8.0, containing 10 mM magnesium acetate and 20 mM ammonium acetate for 1 h at 37°C. Processed RNA samples were diluted 1:3 with acetonitrile + 0.2% (v/v) acetic acid, centrifuged for 10 min at 21 000 × *g*, at 22°C to remove any precipitate, and transferred to glass microvials. Samples (5 μl) were analyzed by a Thermo Ultimate 3000 HPLC coupled with a Q-Exactive Plus mass spectrometer set in both positive and negative ion modes. The samples were separated on a Zic-pHILIC column (5 μm, 150 × 2.1 mm, EMD Millipore) at 0.2 ml/min using the following gradient of mobile phase A (20 mM ammonium carbonate in 0.1% ammonium hydroxide), in B (97% acetonitrile in water): 100% B at 0 min, 40% B at 20 min, 0% B at 30 min for 5 min, then back to 100% B in 5 min, followed by 10 min of re-equilibration with B. The mass spectrometer was calibrated immediately prior to use. Data were analyzed using Thermo Xcalibur 3.0 with ICIS automated peak integration (default settings: smoothing points = 9; baseline window = 40; area noise factor = 2; peak noise factor = 10) followed by manual data curation. In analysis of NP1 treated samples, peak areas were normalized relative to the peak area of the 15N-AMP spike-in.

### Preparation of the ribosome footprint fragment library

*E. coli* BW25113 cells transformed with pBAD33-*itaT*, pBAD-*tacT3* or empty pBAD33 vector were grown overnight in 10 ml of LB medium containing 34 μg/ml chloramphenicol and 1% glucose. The overnight cultures were diluted into 200 ml of MOPS-EZ containing 33 μg/ml chloramphenicol and 1% glucose and grown at 37°C to an OD_600_ of 0.2. The cells were transferred into fresh MOPS-EZ medium without glucose to recover at 37°C for 30 min. Toxin expression was induced with 1 mM arabinose for 15 min. The cells were harvested by rapid filtration, flash-frozen in liquid nitrogen and cryo-lyzed in 650 μl of lysis buffer (10 mM MgCl_2_, 100 mM NH_4_Cl, 5 mM CaCl_2_, 0.4% Triton X-100, 0.1% NP-40 in 20 mM Tris–HCl, pH 8.0) supplemented with 65 U of RNase-free DNase I (Roche), 208 U of SUPERaseIn RNase inhibitor (Invitrogen), and 3 μM GMPPNP (Sigma-Aldrich), as described (25). The cellular debris was precipitated by centrifugation at 20 000 × *g* for 10 min at 4°C. The cleared lysates were diluted with the lysis buffer to an OD_260_ = 0.1. A 220 μl aliquot of the lysates were treated with ∼450 U of MNase (Roche) at 25°C for 1 h, then quenched by the addition of EGTA to 5 mM. The monosomal fraction was purified using centrifugation in a 10–40% sucrose gradient. The RNA purification and DNA library preparation for deep sequencing were carried out as described (25).

### RiboSeq data analysis

The sequencing data were analyzed using the pipeline developed by Mohammad *et al.* (26) (github.com/greenlabjhmi/2018_Bacterial_Pipeline_riboseq) and custom python and R scripts from our previous study (16) (https://github.com/bikdm12/AtaT2-ribo-seq). In brief, the raw reads were quality filtered and trimmed with Skewer 0.2.2 (27). Reads with lengths that did not fall within the 15–40 nt range were discarded. Several consecutive mapping steps were conducted using Bowtie 1.2.2, restricting the number of mismatches to two (28). The footprints that matched tRNA or rRNA sequences were removed from the dataset. The remaining reads were aligned to the reference *E. coli* MG1655 genome (RefSeq accession number NC_000913.2).

To obtain the ribosome density values, we calculated the number of 3′ ends of reads in every genome position and divided this value by the total number of uniquely mapped footprints (in millions). The ORFs with lengths less than 170 bp and the average ribosome density per codon <1 were discarded. As the ribosome density near the start and stop codons are known to be biased, the first 15 and 7 N and C-terminal codons in the ORFs, respectively, were not considered in the downstream analysis (26). The ribosome density was converted to the pause score (PS) by normalizing the density in every position by the average density on that ORF. To accurately calculate the position of the ribosomal A site, we analyzed the average ribosome density for all ORFs aligned at the start and the stop codons (Supplementary Figure S1). The mean pause scores (MPS) for codons and amino acids were obtained by averaging scores on all nucleotides corresponding to the codon (or amino acids) on an ORF and then averaging the resulting values across all ORFs in the analysis.

### Phylogenetic analysis

A previous study of GNAT toxins evolution was performed on the 2005 version of the RefSeq database (15). To collect the dataset for phylogenetic analysis, we first constructed the profile HMMs for the type II GNAT toxins using data from the previous study (15). We excluded sequences with lengths that did not fall within the 150–200 amino acid range from the alignment and realigned the remaining sequences using MUSCLE with defaults parameters (29). The resulting alignment was used to construct the profile HMM utilizing the HMMbuild program from the HMMER package (http://hmmer.org/).

The RefSeq database (30) was downloaded in May 2021 as FASTA format containing every CDS product from all bacterial genome assemblies. The database was then searched with constructed toxin HMM profile using HMMsearch with an *E*-value cut-off 1e–4.

The resulting sequences were clustered using MMseqs2 (31) in connected component mode with the identity cut-off of 60% to obtain the representative set of sequences. This set was then used to construct the sequence similarity network *via* the EFI-EST web server (32). The network was visualized using Cytoscape (33). Edges of the network represent blast scores above 120. The resulting network was additionally clustered using MCL algorithm as implemented in the Clustermaker plugin for Cytoscape (34). For further analysis, two clusters were selected: the largest community containing all GNAT proteins encoded along with the known antitoxin genes and a smaller distinct cluster as an outgroup for phylogenetic tree rooting. Functional annotation of proteins encoded adjacent with the GNAT proteins was performed using Conserved Domains Database (CDD) *via* Batch CD-Search web service (35).

Selected 1238 sequences with the length within 150–245 amino acids were aligned using MUSCLE with a gap open penalty of –5 and a gap extend penalty of –1. Alignment underwent minor manual correction and trimming of the highly variable terminal portions. Columns containing 90% or more gaps were removed with Clipkit (36). Finally, the maximum likelihood phylogenetic tree was built using RAxML with LG substitution matrix and gamma-distributed evolutionary rates (37). Branch support was quantified using Transfer Bootstrap Expectation (TBE) (38) as implemented in ‘BoosterWeb’ tool (https://booster.c3bi.pasteur.fr).The resulting tree was visualized using iTOL (39).

## RESULTS

### GNAT toxins display different aminoacyl-tRNA target preferences *in vitro* and *in vivo*

**Figure 1.**
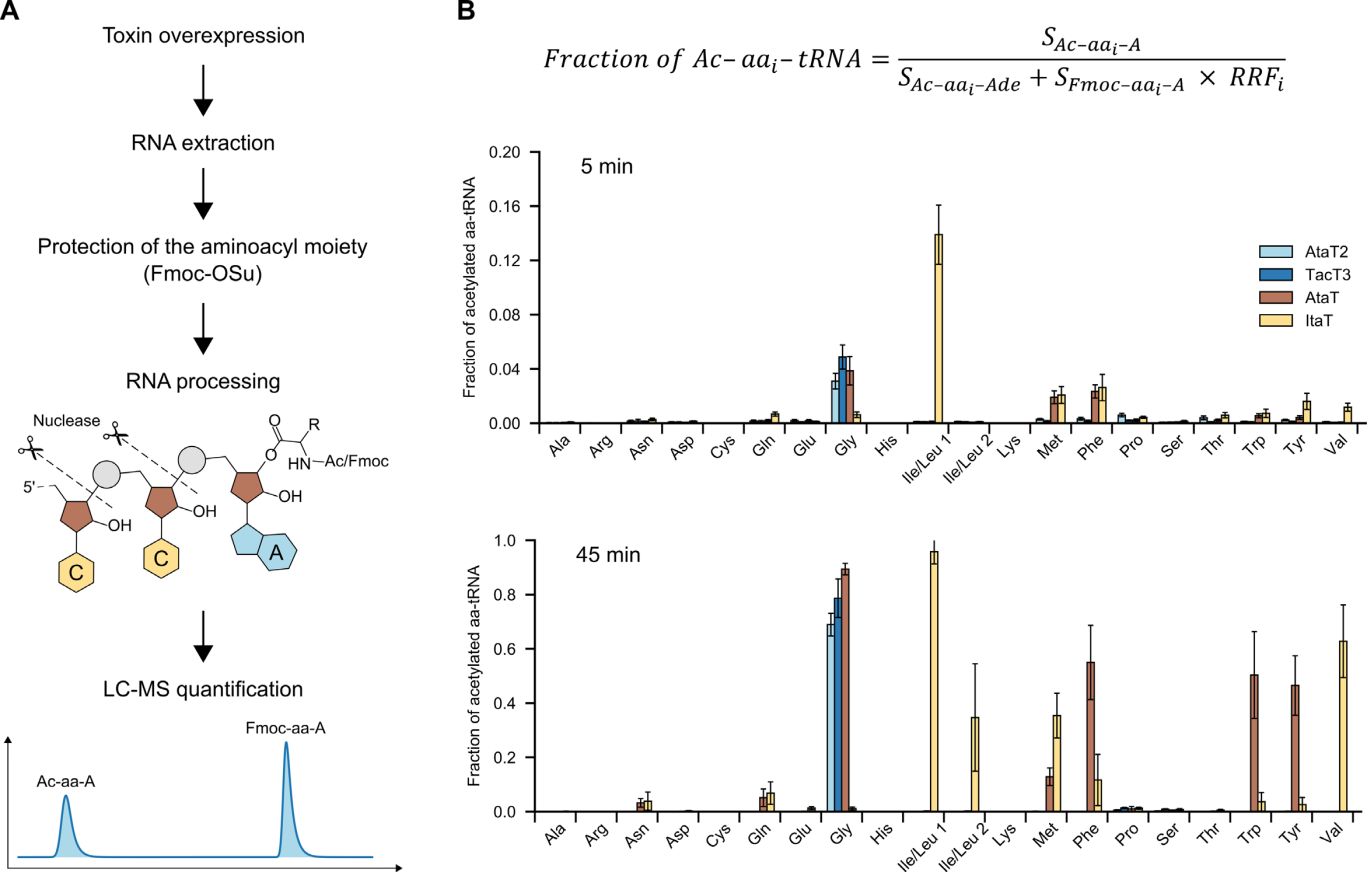
GNAT toxins acetylate different sets of aminoacylated tRNAs. (**A**) Schematic outline of experimental design. tRNA extracted from toxin-overexpressing cells was treated with Fmoc-OSu to stabilize the unmodified aminoacyl moieties and digested by RNase I to generate the Acetyl- and Fmoc-derivatives of aminoacylated 3′-terminal adenosine of aa-tRNAs. The resulting derivatized aa-A were quantified by LC/MS. (**B**) Fractions of acetylated aminoacyl-tRNAs isoacceptors in cells expressing AtaT, ItaT, TacT3, and AtaT2 toxins. Fractions of each Ac-aa-A were calculated using the formula shown above the graph. S, the area under the peak of eluted ion; RRF_i_, the relative response factors experimentally determined for Fmoc derivatives (see Materials and Methods and Supplementary Table S5). Analysis was performed for 5 min (top) and 45 min (bottom) time points of toxin induction. Error bars show the standard deviation values obtained in three independent experiments.

To characterize GNAT toxin target specificities, we first investigated how the expression of four previously studied GNAT toxins, AtaT, AtaT2, and ItaT from *E. coli* and TacT3 from *Salmonella*, modifies the intracellular pool of aminoacyl-tRNAs (aa-tRNAs). For each *E. coli* BW25113 tRNA, we quantified the fraction of acetylated *vs*. non-acetylated aa-tRNA after expression of plasmid-borne GNAT toxin genes for 5 and 45 min (Figure 1A). RNA was isolated, the hydrolyzable ester bond in unmodified aa-tRNAs was stabilized by treatment with Fmoc *N*-hydroxysuccinimide ester (Fmoc-OSu), and the samples were digested with RNase I. Complete RNase I digestion of aa-tRNAs generates aminoacyl-adenosine (aa-A) molecules derived from the 3′-end of tRNAs with unique molecular masses identifiable *via* LC/MS (Figure 1A). The differences in ionization efficiencies for Fmoc-modified and acetylated aa-As were corrected using experimentally determined relative response factors (RRF) (Supplementary Table S5).

Expression of AtaT2 and TacT3 led to acetylation of Gly-tRNA^Gly^ exclusively (Figure 1B, Supplementary Tables S6 and S7). The acetylated fraction of Gly-tRNA^Gly^ approached 70–80% at the 45 min timepoint. While narrow specificity of AtaT2 is in agreement with our previously published results (16), the data show that *in vivo*, TacT3 has a much narrower specificity than reported previously based on *in vitro* analysis (14).

Induction of expression of AtaT or ItaT led to acetylation of multiple aa-tRNAs, corroborating the previously published *in vivo* results (20,21) but differing from the *in vitro* data (15,17). In addition to Gly-tRNA^Gly^ AtaT acetylated several aa-tRNAs charged with hydrophobic amino acids, including Trp-, Tyr-, Phe- and Met-tRNAs (Figure 1B, Supplementary Tables S6 and S7).

Five minuntes post-induction, ItaT showed activity toward aa-tRNAs charged with hydrophobic amino acids with a strong preference toward Leu-/Ile-tRNAs (Figure 1B, Supplementary Tables S6 and S7). At a later timepoint, the acetylation of Val- and Met-tRNAs was markedly increased. HPLC can partially resolve the modified leucyl- and isoleucyl-adenosine derivatives, allowing for deconvolution of these two co-eluted peaks within the chromatogram (Supplementary Figure S2). When compared to the control samples, induction of ItaT clearly results in the complete disappearance of only one of these peaks (Supplementary Figure S2, bottom right-hand panel). As leucine and isoleucine are structural isomers, MS analysis cannot differentiate them, precluding identity assignment for these peaks. We conclude that ItaT possesses a clear preference to either leucyl- or isoleucyl-tRNA and that only one of the two is the primary target for the toxin.

Overall, our data demonstrate that *in vivo* TacT3 and AtaT2 have a strong preference for Gly-tRNA^Gly^, whereas AtaT and ItaT exhibit a broader specificity with a bias toward Gly-tRNA^Gly^ and Leu/Ile-tRNA^Leu/Ile^, respectively. All four toxins show partially overlapping target specificities and their patterns of acetylated aa-tRNAs deviate from those reported *in vitro* (14,15,17).

### GNAT toxins preferentially target translation elongation

**Figure 2.**
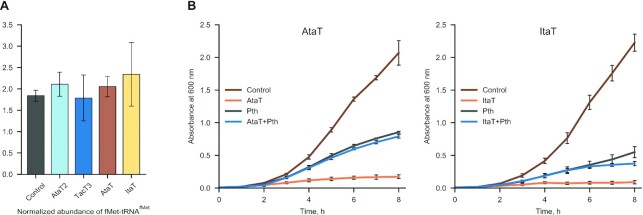
Expression of GNAT toxins does not abolish translation initiation. (**A**) Relative fMet-tRNA^fMet^ abundances in control and toxin-expressing cells after 45 min of induction taken from the experiment shown in Figure 1B. Peak areas of fMet-Ade ([M + H]^+^ at *m/z* 427.13) were normalized to that of Fmoc-Pro-Ade ([M + H]^+^ at *m/z* 587.22) in each sample. (**B**). Peptidyl tRNA Hydrolase (Pth) neutralizes the toxicity of AtaT and ItaT overexpressed in *E. coli*. Each panel shows the growth curves of bacteria expressing toxin or Pth alone or together with the toxin with Pth, using an empty vector as control. Data points show the mean of three biological replicates ± standard deviation.

MS analysis does not discriminate between acetylated elongator and initiator methionyl-tRNAs after treatment with RNase I. Since it has been reported that AtaT and ItaT target the initiator Met-tRNA^fMet^*in vitro* (17,20), we hypothesized that the acetylated Met-tRNA observed in ItaT and AtaT producing cells (Figure 1B) could be the result of acetylation of unformylated initiator Met-tRNA^fMet^. To test this hypothesis and overcome the limitations of MS analysis, we first examined the LC/MS chromatogram for the presence of mass-ions corresponding to *f*Met-tRNA*^f^*^Met^-derived *f*Met-adenosine (fMet-A). We speculated that if the initiator Met-tRNA*^f^*^Met^ were the main target of AtaT, the abundance of *f*Met-A would decrease as a consequence of toxin induction. Since none of the studied toxins significantly acetylate Pro-tRNA^Pro^, we used Fmoc derivatized Pro-A from proline tRNA to normalize *f*Met-A abundances in each sample. The relative amount of *f*Met-A did not differ significantly between the empty vector control and the RNA sample from GNAT toxin-expressing cells (Figure 2A, Supplementary Table S8), in agreement with earlier observations (20). This result indicates either that ItaT and AtaT do not target Met-tRNA*^f^*^Met^, or that the concentration of initiator Met-tRNA*^f^*^Met^ is maintained constant, even when these GNAT toxins are overproduced, by an unidentified compensatory mechanism.

Previously, it was shown that, when co-expressed with the TacT toxins, the essential bacterial enzyme peptidyl-tRNA hydrolase (Pth) removes *N*-acetyl-amino acids from aa-tRNAs corrupted by the toxin, thereby enabling tRNA recycling and protecting *Salmonella* from TacT toxicity (13,14). While *N*-blocked elongator aa-tRNAs are substrates for Pth, the enzyme does not hydrolyze *N*-formylated or *N*-acetylated initiator Met-tRNA*^f^*^Met^ (40,41). We therefore reasoned that if AtaT or ItaT target elongator aa-tRNAs, as TacT toxins do, their toxicity should be neutralized by Pth. To test this hypothesis, we expressed AtaT and ItaT in *E. coli* alone or together with Pth and monitored bacterial growth by optical density. Both ItaT and AtaT strongly inhibited bacterial growth (Figure 2B). While Pth overexpression is toxic to *E. coli* to some extent, co-expression of AtaT or ItaT did not lead to further growth inhibition (Figure 2B). We ensured that the pBAD30-*ataT* and pBAD30-*itaT* vectors retained toxicity in an inducer-dependent manner in strains carrying the *p*th expressing plasmid pCA24N-*pth* (Supplementary Figure S3A) and that GNAT toxicity was not counteracted by expression of control genes from the pCA24N vector (Supplementary Figure S3B). These controls confirm that Pth specifically counteracts the activity of toxins tested, providing further evidence that corruption of initiator Met-tRNA*^f^*^Met^ is not the cause of AtaT and ItaT toxicity. Thus, both the AtaT and ItaT toxins affect translation at the elongation step.

### GNAT toxins acetylate all isoacceptor tRNAs of their preferred amino acid targets

**Figure 3.**
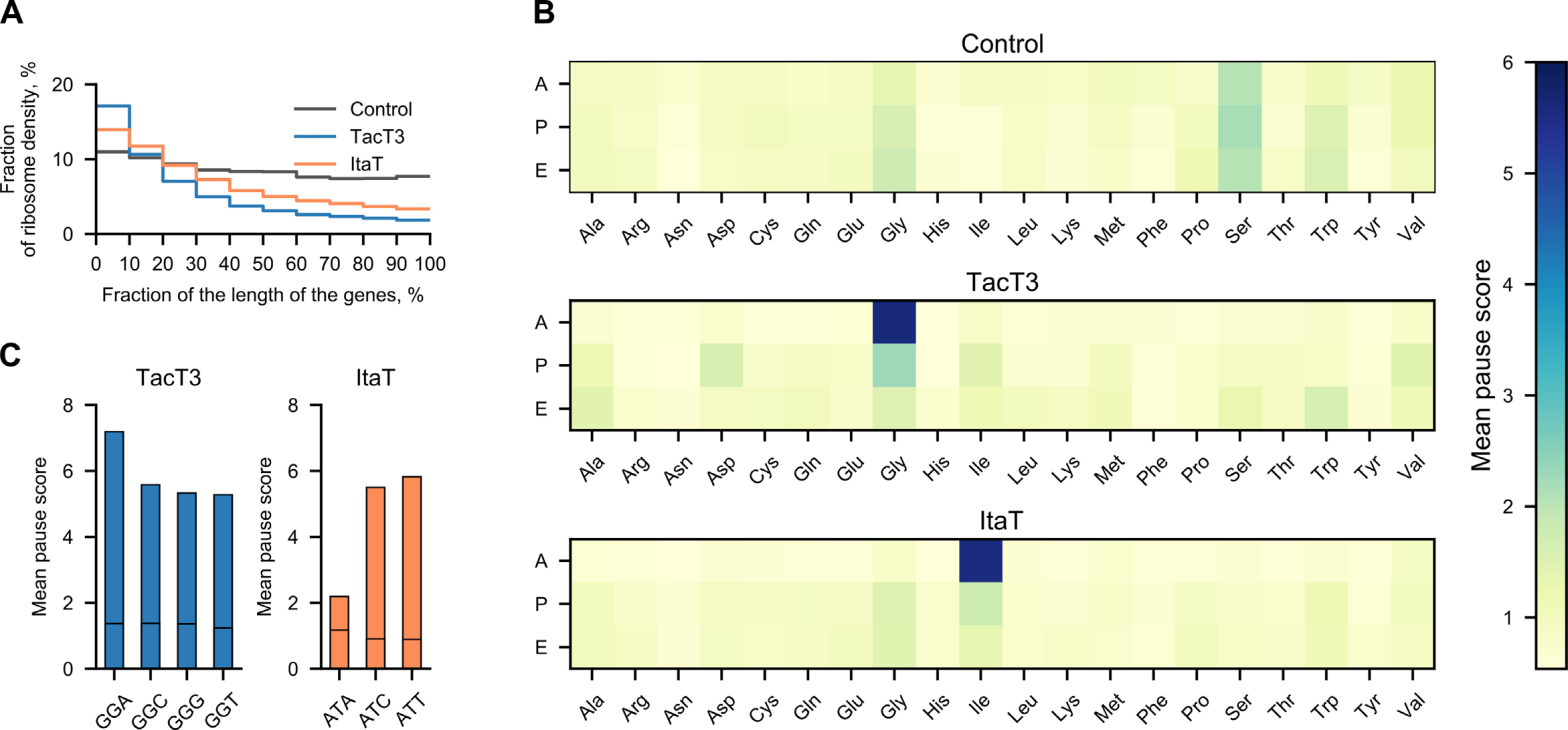
GNAT toxin activity causes ribosome stalling at specific codons. (**A**) Distribution of ribosomes over ORFs in cells harboring pBAD, pBAD-*tacT3*, and pBAD-*itaT*. Each coding region was divided into ten equal bins from 5′- to 3′-end, and the fraction of ribosomes mapped to each bin was calculated. The plot shows the geometric means of the ribosome density for each bin. (**B**) Heatmaps of MPS (the mean ribosome density at a particular codon normalized to the mean density across the given ORF) for each codon located in A, P, and E sites of ribosomes, during TacT3 or ItaT intoxication, or in a negative control sample. (**C**) Mean pause scores for individual Gly and Ile codons located in the ribosomal A-site in the cells expressing TacT3 and ItaT toxins, respectively. Black horizontal lines represent mean pause scores in cells with an empty pBAD vector.

To experimentally assess isoacceptor biases and gain further insights into the physiological consequences of GNAT toxin activities, we performed ribosome profiling of GNAT toxin-overexpressing bacteria. This approach examines the distribution of ribosomes on mRNAs at single-codon resolution (42). For this analysis, we chose ItaT and TacT3 as examples of broad- and narrow-specificity toxins, respectively. We induced expression of toxin genes for 15 min, isolated ribosome-bound mRNA, and treated it with micrococcal nuclease to generate ribosome-protected RNA fragments that were then subjected to deep sequencing. We next mapped the resulting ribosome footprints to the genome to infer ribosome distribution. As expected, the ribosomes were relatively evenly distributed over the entire length of open reading frames (ORFs) in the empty vector control sample. By contrast, in cells expressing the toxins, the ribosome density was significantly higher at the beginning of ORFs, indicating translation inhibition (Figure 3A).

For each genome position, we determined the pause score (PS), calculated as the ribosome density at a particular nucleotide normalized to the mean density across the corresponding ORF. Next, we calculated the mean pause score (MPS) across all ORFs for codons located in the A-, P-, and E-sites of the ribosome. A slight increase in the MPS was observed in all three ribosomal sites for all serine and glycine codons; however, this is a common artifact of ribosome profiling experiments (26), which was also observed in the control sample (Figure 3B). In cells expressing TacT3, a sharp increase in the MPS was uniquely detected for glycine codons located in the A-site of the ribosome, consistent with the LC/MS data. Of the four glycine codons, MPSs were similarly high for GGC, GGU, and GGG and slightly higher for the GGA codon (Figure 3C). These data indicate that TacT3 targets all three Gly-tRNA^Gly^ isoacceptors with similar efficiencies. In cells overexpressing ItaT, a substantial increase in the MPS was observed specifically for isoleucine codons (Figure 3B). This result discriminates the ItaT substrate preference further than the LC/MS approach and shows that the isoleucyl-tRNAs but not leucyl-tRNAs are the primary targets of ItaT. High MPSs were observed for the AUU and AUC isoleucine codons, with a substantially lower MPS for the AUA codon (Figure 3C). Since in *E. coli*, the AUU and AUC codons are recognized by a single Ile-tRNA(GAU), while the rare AUA codon is decoded by a minor Ile-tRNA(k2CAU), we conclude that ItaT has a preference toward Ile-tRNA(GAU). Thus, despite the broad substrate specificity of the ItaT toxin, its primary physiological target is Ile-tRNA^Ile^. Altogether, our results show that for the two GNAT toxins tested, all isoacceptors of the targeted aminoacyl-tRNA group are substrates for acetylation.

### The narrow-specificity GNAT toxins evolved from a promiscuous common ancestor

**Figure 4.**
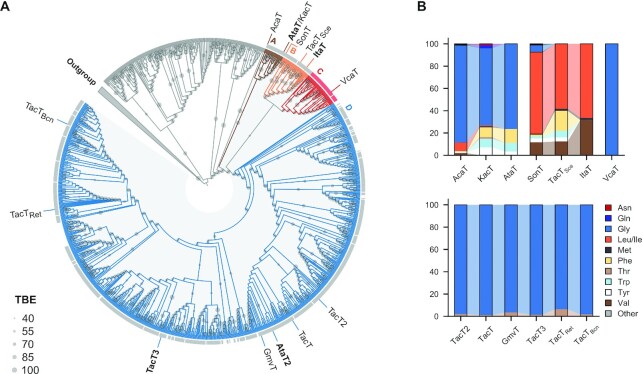
GNAT toxins evolve toward narrow substrate specificity. (**A**) The cladogram of GNAT toxin homologs. Transfer Bootstrap Expectation (values ≥ 40%) is shown as grey circles. The segmented colored outer ring indicates antitoxin genes adjacent to the GNAT toxin genes: grey, canonical antitoxins containing DUF1778; and red, novel antitoxins containing DUF4065. Clades are colored and labeled with a letter. Toxins experimentally studied in this work are indicated outside of the ring. (**B**) LC/MS determined tRNA target specificity of GNAT toxins. Stacked bars represent the total LC/MS signal for all N-acetylated aa-tRNAs detected, grouped by amino acid, for cells expressing the indicated GNAT toxins. Toxins were expressed in exponential cultures of *S*. Typhimurium (TacT, TacT2, TacT3) or *E. coli* (AtaT, KacT, AcaT, ItaT, SonT, VcaT, TacT_Sce_, GmvT, TacT_Bcn_, TacT_Ret_) for 4 h, RNA was extracted and hydrolyzed by Nuclease P1, and the resulting acetylated aminoacyl-adenylates (Ac-aa-A) were quantified by LC/MS.

We hypothesized that the substrate specificity of GNAT toxins might reflect their evolutionary trajectory. To build a comprehensive phylogenetic tree of GNAT toxins, we carried out a bioinformatic search for putative TA type II GNAT toxins in the RefSeq database. The profile Hidden Markov Model used for the database search was constructed using the sequences of acetyltransferase toxins identified in our previous study (15). The resulting dataset was clustered at 60% identity, and the representative sequences were used to build a sequence similarity network (Supplementary Figure S4). GNAT toxins are known to be associated with the DUF1778 (COG4453) protein family antitoxins (1). Therefore, we examined the dataset for the presence of a DUF1778 domain in protein-coding genes located immediately upstream and downstream of the acetyltransferase genes. All TA pairs identified in the analysis clustered to the largest group (Supplementary Figure S4, blue nodes). Next, we built a phylogenetic tree of proteins from this group to further investigate the diversity of the GNAT toxins. The second-largest cluster was used as an outgroup to root the tree. The GNAT-DUF1778 gene pairs form a distinct ‘true TA clade’ on the tree, suggesting a common ancestor to these TA systems (Figure 4A, shaded in grey, and Supplementary Figure S5).

As can be seen from Figure 4A, a distinct group of GNATs forming a separate clade C is not associated with DUF1778 antitoxins. We noticed that acetyltransferases from this clade are associated with proteins of unknown function containing a DUF4065 domain, which was recently proposed to act as a universal antitoxin (43) (Figure 4A). As antitoxin gene shuffling between different type II TAs occurs relatively frequently (44,45), we hypothesized that DUF4065-GNATs form a new toxin–antitoxin pair. To test if clade C GNATs are toxic and are part of functional TA pairs, we selected two adjacent genes from *Varibaculum cambriense* that we named VcaA-VcaT. To make sure that the putative toxin is expressed and retains toxicity we created a 3xFLAG fused version of VcaT. Expression of VcaT alone resulted in cell growth inhibition, while co-expression of VcaA alleviated the toxic effect of VcaT (Supplementary Figure S6A). It is worth noting that the amount of VcaT produced in the cells expressing *vcaT* and *vcaAT* was comparable (Supplementary Figure S6B). Thus our data confirm that clade C encodes functional novel TA pairs.

To relate the phylogenetic analysis of the toxins to their biochemical properties, we located the broad (AtaT/ItaT) and narrow (TacT3/AtaT2) specificity toxins within the phylogenetic tree. Unexpectedly, we found that toxins exhibiting relaxed specificity, AtaT and ItaT, belong to different clades: AtaT is a member of the basal clade A, whereas ItaT belongs to clade B and evolved further from the GNAT toxin common ancestor. Glycine-specific TacT3 and AtaT2 belong to two sister subclades within a distant clade D (Figure 4A).

To assess whether the proposed tree topology reflects the aa-tRNA target specificity of toxins, we selected ten additional GNAT toxins from across the tree and analyzed the profile of acetylated aa-tRNAs generated by these acetyltransferases by LC/MS. To reveal all aa-tRNA species susceptible to modification, GNAT expression was carried out for 3 h (Supplementary Table S9). As expected, toxins from clade A, namely *E. coli* AtaT, *Klebsiella pneumoniae* KacT, and *Aeromonas caviae* KAM329 AcaT, acetylated very similar sets of aa-tRNAs charged with glycine and hydrophobic amino acids (Figure 4B). In clade B, plasmid-borne toxins TacT_Sce_ from *Salmonella enterica* serovar Cerro and SonT from *Shewanella oneidensis* both displayed similar, although slightly more diverse profiles of acetylated aa-tRNA species as their close homolog ItaT, with a preference for Ile/Leu-tRNAs and Val-tRNA. Clade C VcaT acetylated predominantly Gly-tRNA^Gly^ (Figure 4B and Supplementary Table S9). Five clade D toxins (Figure 4A), including TacT and TacT2 from *S*. Typhimurium (14), *Shigella sonnei* GmvT (19), and two previously uncharacterized toxins – TacT_Bce_ from *Burkholderia cenocepacia* and TacT_Ret_ from *Rhizobium etli* bv. mimosae, had Gly-tRNA^Gly^ as an almost exclusive target. With each of these toxins, a small signal corresponding to acetylation of Thr-tRNA^Thr^ was also detected. It should be noted that in several negative control (empty vector) samples, we also observed a trace signal matching the expected mass of acetylated Thr-tRNA^Thr^. These results suggest that either a contaminating compound of the same mass is present in *E. coli* extracts or that there exists an unidentified mechanism for the production of acetylated Thr-tRNA^Thr^. As aa-As generated by nuclease treatment of glutamyl-tRNA and *N*-acetyl-seryl-tRNA are isobaric ([M + H]^+^ ion at *m/z* 476.1057), we assessed the presence of *N*-acetyl-serine in the samples using LC/MS analysis of RNA processed by Pth, as described previously (14). Pth is known to specifically cleave amino acids from *N-*blocked aminoacyl-tRNAs, including *N*-acetylated aminoacyl-tRNA, without releasing unmodified amino acids (40,41). LC/MS of glutamic acid and *N-*acetylserine chemical standards allows for discrimination between these two amino acids via accurate measurement of retention times. In all Pth-processed RNA samples, we did not detect any acetylated serine generated by the GNAT toxins investigated (Supplementary Figure S7).

Together, our data show that toxins with distinct specificities are located on different clades within the phylogenetic tree. We propose that GNAT toxins that exhibit a narrow specificity toward Gly-tRNA^Gly^ and populate the largest clade D have evolved from a more promiscuous ancestor.

## DISCUSSION

In this work, we combined the identification of *in vivo* substrate specificity and comprehensive phylogenetic analysis to elucidate the evolutionary trajectory within the type II TA systems GNAT toxins family. Initially, bioinformatic prediction of novel GNAT toxins was based on their association with DUF1778 proteins harboring a ribbon-helix-helix (RHH) DNA-binding domain typical for antitoxins from the type II TA systems (1). Our analysis reveals a distinct clade within the phylogenetic tree (designated as ‘true TA clade’) that contains DUF1778-associated GNATs (Figure 4A, light gray-shaded sector), suggesting that this TA pair emerged in a single evolutionary event. The tree also contains several putative acetyltransferase genes, part of TA-like bicistronic loci, located outside the ‘true TA clade’. However, the short ORFs preceding these acetyltransferase genes are divergent and lack any predictable DNA-binding domains characteristic of classical type II antitoxins. We speculate that despite sharing a common ancestry with GNAT toxins from the type II TA systems, acetyltransferases external to the ‘true TA clade’ either form TA pairs with yet unidentified antitoxins or may have evolved different physiological functions.

Within the ‘true TA clade’, we identified a distinct group of GNAT toxins associated with DUF4065 proteins. Our data indicate that this DUF4065-GNAT pair constitutes a new family of toxin–antitoxin systems, which evolved from the ancestral TAs by substituting the DUF1778 antitoxin gene for a different one. The DUF4065 proteins have been previously identified in some predicted (1) and validated (43,46–47) TA systems. SocAB, a type VI TA module of *Caulobacter crescentus*, represents an example of a characterized TA with a DUF4065 antitoxin. In contrast to type II antitoxins, which inactivate their cognate toxins by sequestration, the SocA antitoxin interacts with both the SocB toxin and with the N-terminal domain of the ClpX protease, thus promoting efficient toxin degradation (46). Whether VcaA-VcaT functions as a type VI TA remains to be determined.

Our comprehensive LC/MS profiling of toxins' *in vivo* acetylation activity demonstrates that the representatives of the most basal clade A possess relaxed target specificity. Toxins from a relatively small clade B also acetylate multiple aminoacylated tRNAs, which, however, are different from the set acetylated by clade A toxins. Toxins belonging to clades C and D exhibit narrow substrate specificity and modify almost exclusively Gly-tRNA^Gly^. Together, clades C and D comprise most of identified GNAT toxins diversity, which indicates that the narrow specificity may provide an evolutionary advantage over the relaxed specificity.

Our data suggest that while evolutionarily ancient GNAT toxins associated with type II TA systems had a less stringent preference for aa-tRNA targets, subsequent evolution has driven these toxins toward a more refined target specificity. Promiscuity, or the ability of an enzyme to catalyze reactions not essential for their biological function, plays a key role in evolution (48). It is not surprising that upon their appearance, due to a switch of function, ancestral GNAT toxins possessed relaxed substrate specificity towards aa-tRNAs. Strict substrate specificity is believed to be shaped by both negative and positive selection: the positive selection tends to optimize the interaction between active-site and the reaction's transition-state; the negative selection aims to prevent reactions with competing off-target substrates (49). The results of ribosome profiling demonstrate that the apparent broad specificity of the ItaT toxin revealed in the MS profiling experiment is rather redundant as its primary physiologically relevant target is Ile-tRNA^Ile^. One can speculate that once the ribosomes have stalled on isoleucyl codons, the translation is halted; thus the availability of other aminoacylated tRNA has little if any additional effect on cell physiology.

From our phylogenetic reconstruction, it is hard to conclude whether the narrow specificity toward glycyl-tRNA arose independently in the ancestors of clades C and D toxins or the last common ancestor of clades B, C, and D evolved the specificity toward glycyl-tRNA. In the latter scenario, toxins from clade B should have broadened and switched their specificity with a clear preference toward isoleucyl-tRNA acetylation. Why narrow-specificity toxins evolved to only target glycyl-tRNAs remains to be determined. Targeting specific aa-tRNAs may result in novel advantageous biological outcomes. The disruption of glycine incorporation into the proteome, or the resulting disturbance of metabolic pathways linked to this amino acid, may confer key advantages which have driven the evolution of these toxins. It is noteworthy that GNAT toxin homologs are almost absent from the Gram-positive *Firmicutes* (Supplementary Figure S5). *Firmicutes* such as *Staphylococcus aureus* rely on Gly-tRNA^Gly^ for peptidoglycan synthesis (50), which could make them more sensitive to inactivating modification of glycyl-tRNAs.

A recent study (22) showed that both the sequence of the acceptor stem of tRNA and the nature of the aminoacyl moiety are crucial for specific recognition of the target by the TacT toxin. Importantly, aminoacyl moieties other than glycine are rejected from the active site of the enzyme due to the bulky side chains (22), and we can hypothesize that this simple but robust mechanism that facilitates high selectivity may be the reason for expansion of glycyl-tRNA-specific GNAT toxins.

## DATA AVAILABILITY

Raw data from RiboSeq experiments are deposited at GEO under accession number GSE148424. Custom python scripts for LC/MS data analysis are available at https://github.com/bikdm12/GNAT_toxins.

## Supplementary Material

gkac356_Supplemental_Files
