# Supplementary material for: GNAT toxins evolve toward narrow tRNA target specificities
Source: Nucleic Acids Res. 2022 May 24;50(10):5807–17. doi: 10.1093/nar/gkac356 (PMC9177977; doi:10.1093/nar/gkac356)
Supplement: gkac356_Supplemental_Files [file gkac356_supplemental_files.zip › Supplementary Materials_Bikmetov-GNATs.pdf]

GNAT toxins evolve toward narrow tRNA target specificities

Dmitry Bikmetov, Alexander M J Hall, Alexei Livenskyi, Bridget Gollan, Stepan Ovchinnikov, Konstantin Gilep, Jenny Y. Kim, Gerald Larrouy-Maumus, Viktor Zgoda, Sergei Borukhov, Konstantin Severinov*, Sophie Helaine* and Svetlana Dubiley*

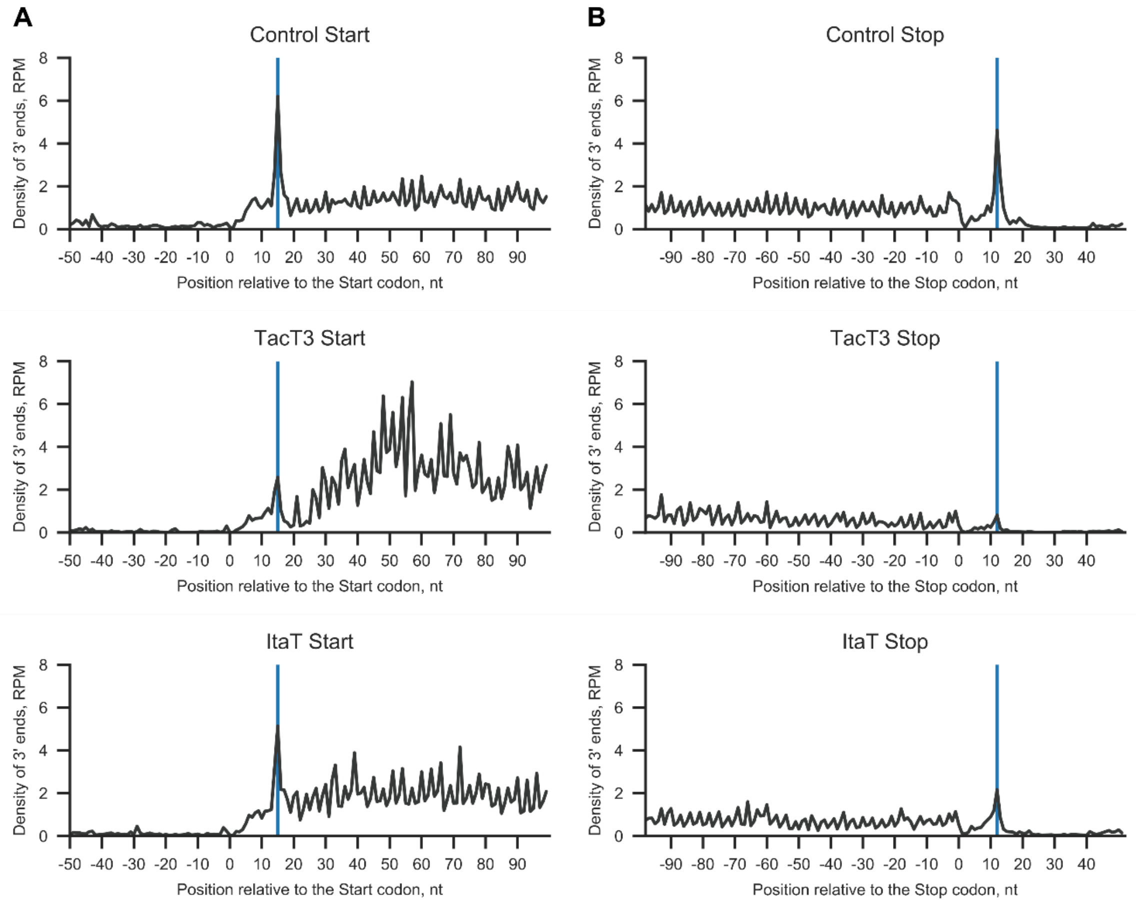

Supplementary Figure 1. Ribosome density plots averaged for all ORFs and aligned at the start or the stop codons (metagene analysis). Zero-point on the horizontal axis corresponds to the first nucleotide of the start codon (A panels) or the stop codon (B panels), respectively. In (A), the first major peak (at 15 nt, marked by the blue vertical line) corresponds to the 3'-ends of footprints with the start codon located in the ribosomal P site. The last major peak in (B) (12 nt, marked by the blue vertical line) corresponds to the 3'-ends of footprints with the stop codon located in the ribosomal A site.

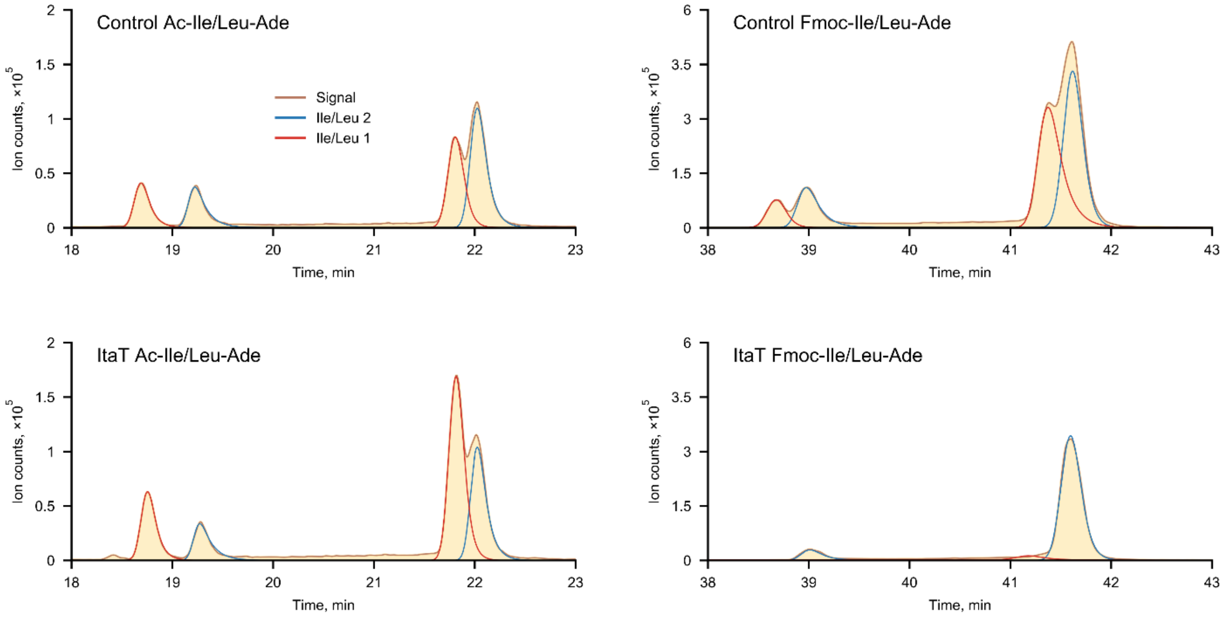

Supplementary Figure 2. Deconvolution of co-eluted peaks in the extracted chromatograms of [MH]+ at *m/z* 423.19 and [MH]+ at *m/z* 603.25 ions corresponding to Ac-Ile-A/Ac-Leu-A and Fmoc-Ile-A/Fmoc-Leu-A, respectively. LC/MS analysis of equal amounts of tRNA extracted from control *E. coli* BW25113 cells harboring pBAD, pre-treated with acetic anhydride (upper left panel) or Fmoc-OSu (upper right panel) and digested with RNase I, or extracted from *E. coli* BW25113 harboring pBAD-*itaT* after 45 minutes of arabinose induction, untreated (lower left panel), or treated with Fmoc-OSu (lower right panel) and digested with RNase I. The pair of peaks on the left and the right of each chromatogram correspond to 3’-Ile/Leu-A and 2’-Ile/Leu-A structural isomers.

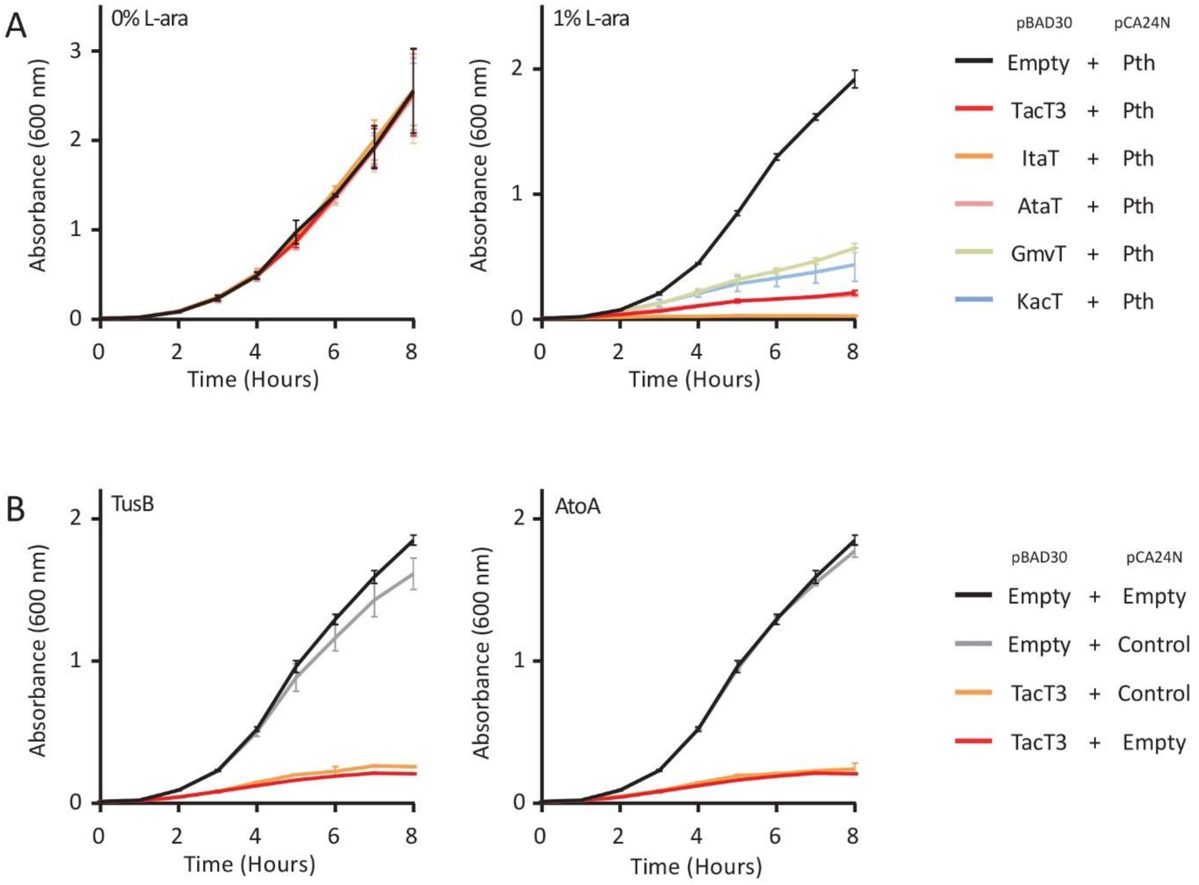

Supplementary Figure 3. A. Bacterial strains co-transformed with pCA24N-pth and pBAD30-GNAT constructs all retains a functional toxin that inhibits growth in response to arabinose induction. Each panel shows growth curves of bacteria with or without induction of toxin expression by incubation with 1% L-arabinose. **B. Expression of *atoA* and *tusB* control genes from the pCA24N vector does not counteract the toxicity of TacT3**. Each panel shows growth curves of *E. coli* co-expressing TacT3 with the control gene as indicated. Data points show the mean of three biological replicates ± standard deviation.

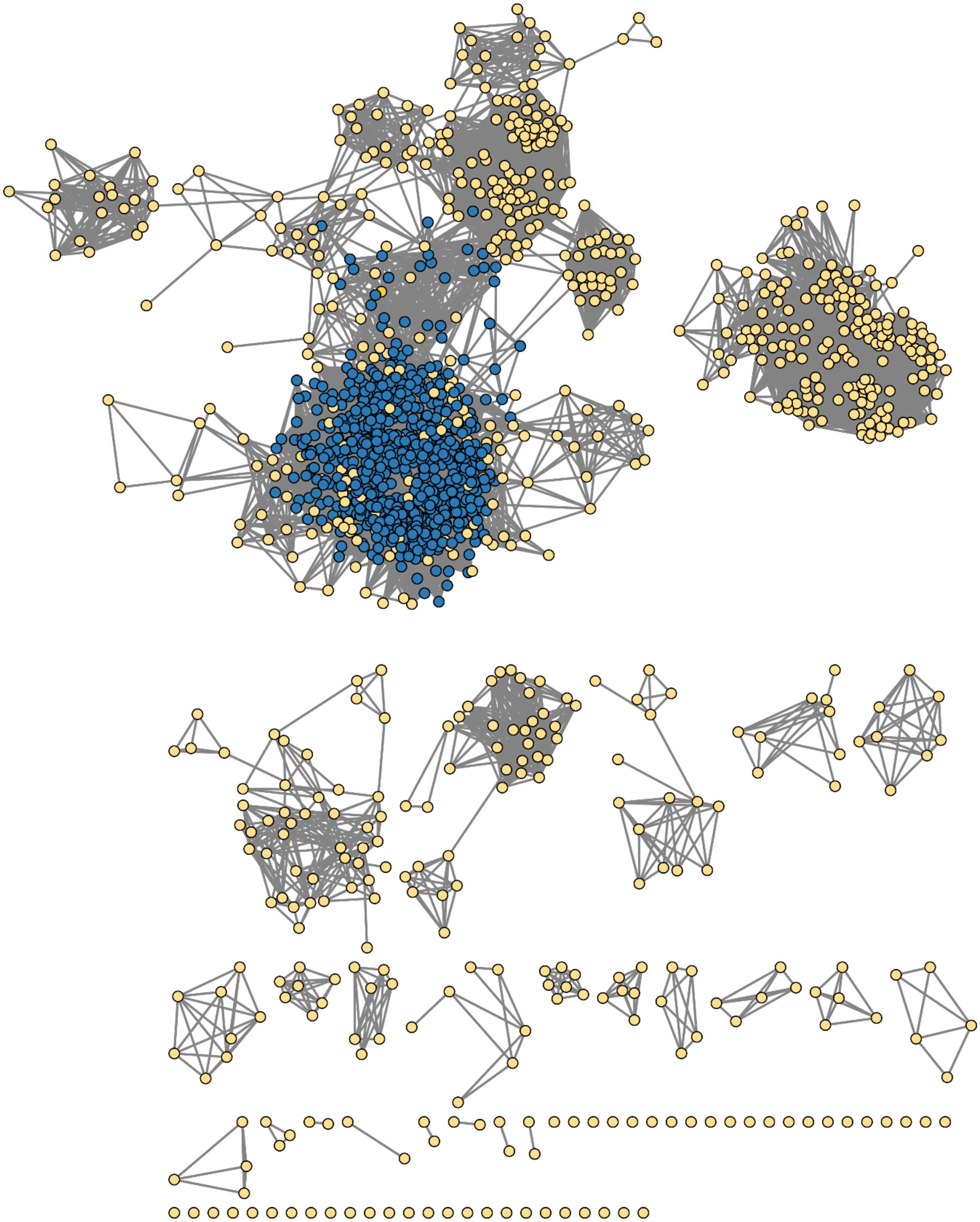

Supplementary Figure 4. Sequence similarity network of GNAT acetyltransferases. Nodes, corresponding to DUF1778-associated GNATs are marked in blue color.

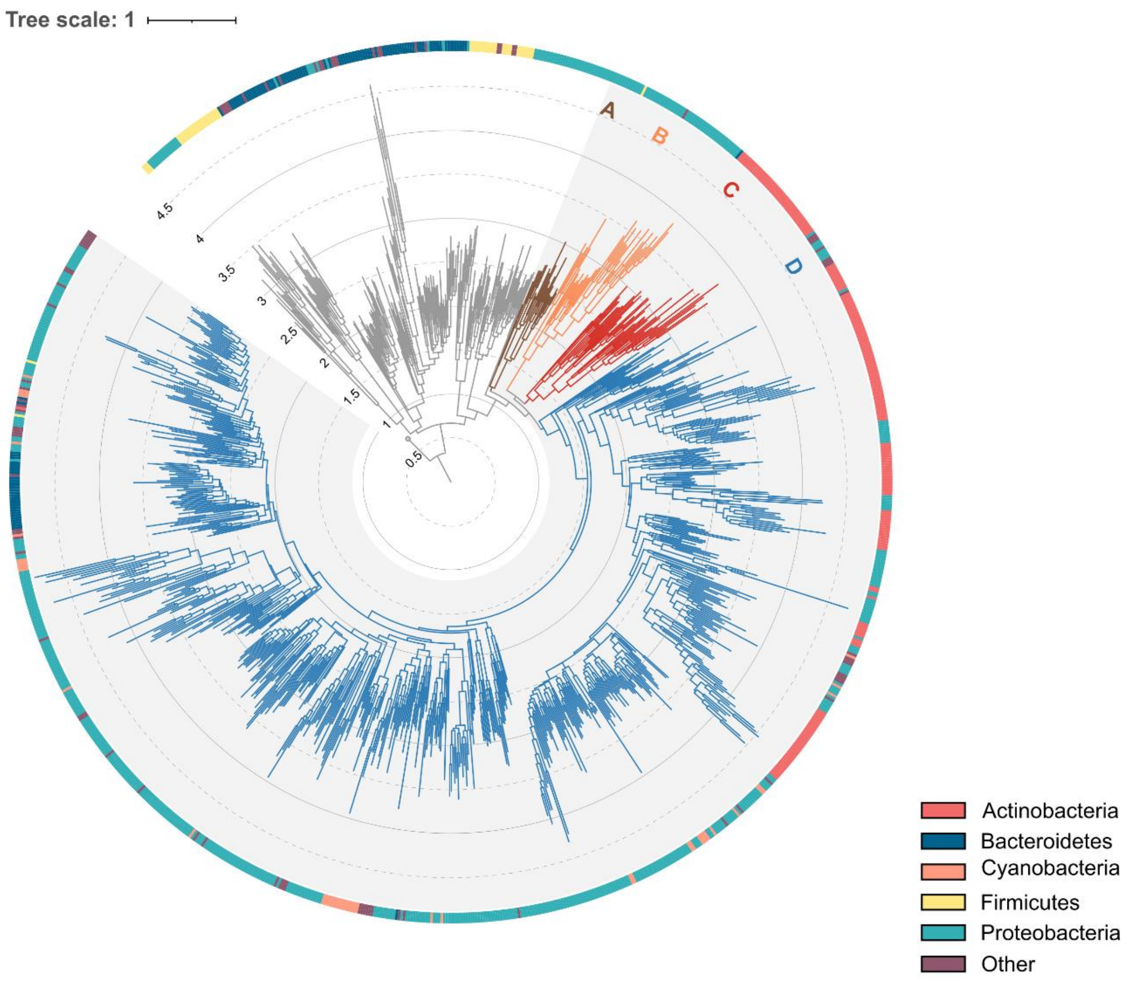

Supplementary Figure 5. Maximum likelihood phylogenetic tree of GNAT toxin homologs. Colored outer ring indicates bacterial phyla. Clade branches are colored and labeled with a letter as in Figure 4A.

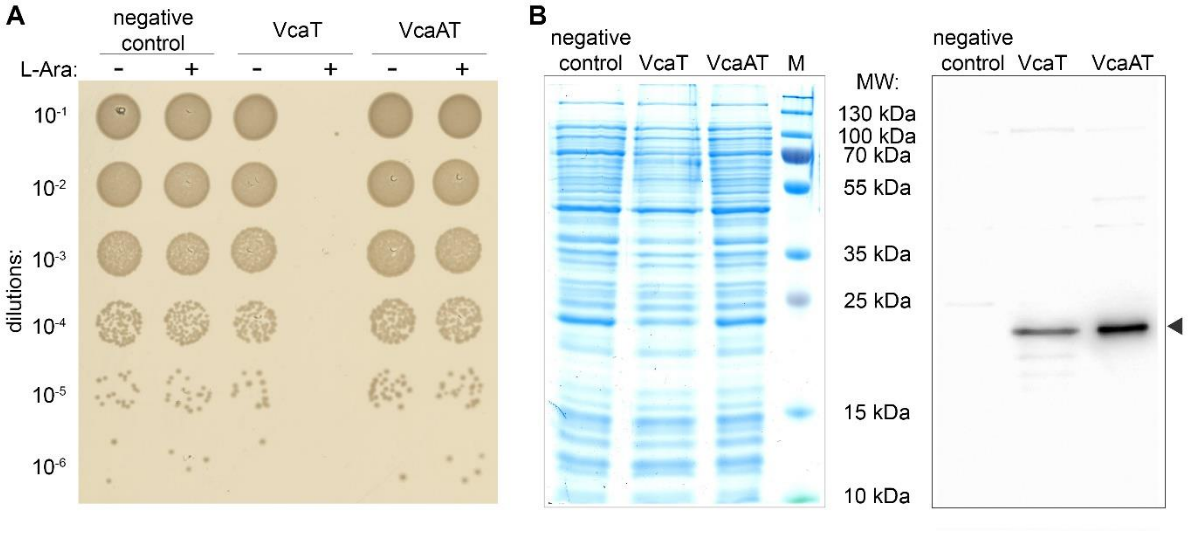

Supplementary Figure 6. DUF4065-GNAT constitutes a novel toxin-antitoxin pair. (A) Aliquots of serial dilutions of cultures of *E. coli* BW25113 cells harboring pBAD33-3xFLAG (as a negative control), pBAD33-*vcaT-3xFLAG*, or pBAD33-*vcaAT-3xFLAG* plasmids grown in the presence (+) or in the absence (-) of arabinose for 60 minutes were spotted on LB agar plates. Results of overnight growth at 37 ˚C are presented. **(B)** SDS-PAGE (left panel) and Western blot (right panel) analyses of whole cell extracts of *E. coli* BW25113 harboring pBAD33, pBAD33-*vcaT-3xFLAG*, or pBAD33-*vcaAT-3xFLAG* 60 minutes post-induction time. The Western blot was developed using anti-FLAG mouse antibody. M – molecular weight markers. The band corresponding to VcaT-F3xFLAG (MW 23 kDa) is marked with a black triangle.

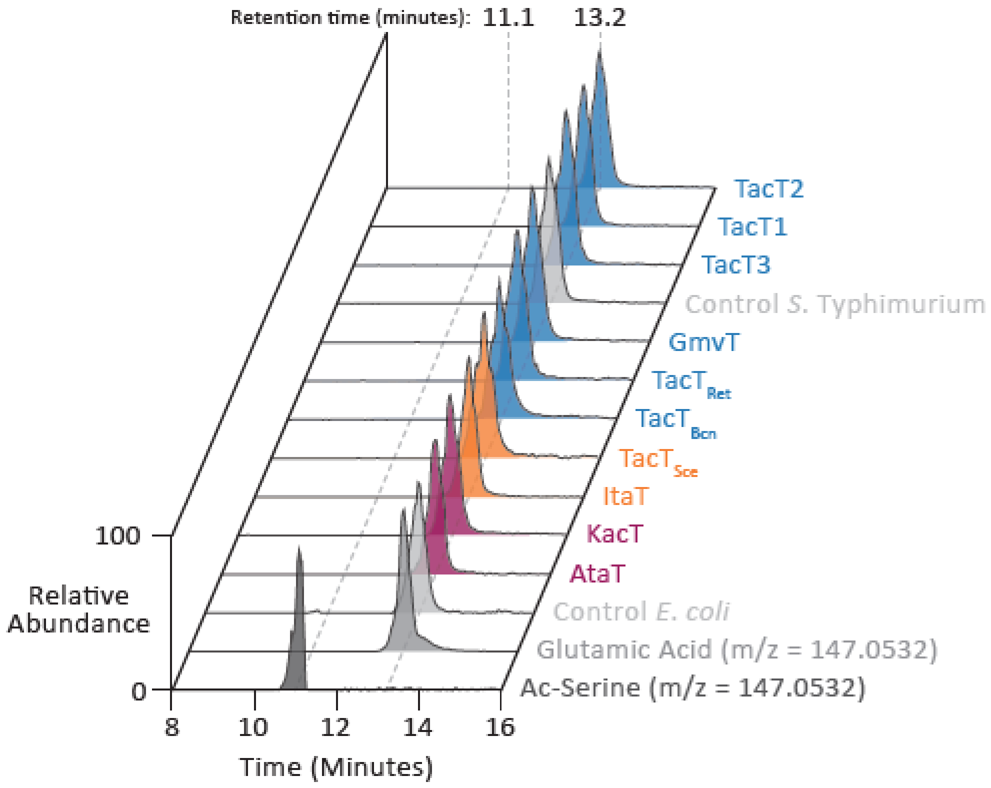

Supplementary Figure 7. GNAT toxins do not acetylate seryl-tRNA. A) RNA from GNAT intoxicated bacteria were treated with peptidyl-tRNA hydrolase (Pth) and the digested samples analyzed by LC/MS. Comparison of extracted ion chromatograms of Pth-digested RNA samples against the chemical standards for N-acetyl-serine and glutamic acid ([MH]+ at *m/z* 147.0532) demonstrates that, for the toxins investigated in this study, acetyl-Ser-tRNA^{Ser} is not a product of GNAT toxin activity.

Supplementary Table 1 | Plasmids

Plasmid	Source/Reference
pBAD30	Guzman *et al*., J Bacteriol. 1995; 177:4121-30
pBAD30-*ataT*	This work
pBAD30-*gmv*T	This work
pBAD30-*itaT*	Wilcox et al., Nucleic Acids Res. 2018 Sep 6;46:7873-7885
pBAD30-*kacT*	This work
pBAD30-*tacT3*	This work
pBAD30-$tacT_{Bcn}$	This work
pBAD30-$tacT_{Ret}$	This work
pBAD30-$tacT_{Sce}$	This work
pBAD33	Guzman *et al*., J Bacteriol. 1995; 177:4121-30
pBAD33-3xFLAG	This work
pBAD33-*acaT*	This work
pBAD33-*ataT2*	Ovchinnikov et al., Nucleic Acids Res. 2020; 48:8617-8625
pBAD33-*itaT*	This work
pBAD33-*sonT*	This work
pBAD33-*tacT1*	Rycroft *et al*., Nat Commun. 2018; 9:1993
pBAD33-$tacT2_{Sen}$	(14) Rycroft *et al*., Nat Commun. 2018; 9:1993
pBAD33-*tacT3*	(14) Rycroft *et al*., Nat Commun. 2018; 9:1993
pBAD33-*vcaT*	This work
pBAD33-*vcaAT-3xFLAG*	This work
pBAD33-*vcaT-3xFLAG*	This work
pCA24-*atoA*	(28) Kitagawa *et al*., DNA Res. 2005; 12:291-299
pCA24-*pth*	(28) Kitagawa *et al*., DNA Res. 2005; 12:291-299
pCA24-*tusB*	(28) Kitagawa *et al*., DNA Res. 2005; 12:291-299

Supplementary Table 2 | Primers

Primer Name	Sequence
AH3.04 AtaT F SalI	GATCGTCGACAGGGAATGTAATAATTTATGGATGATCTGACGATAGAG
AH3.02 AtaT R HindIII	GATCAAGCTTTTAATCGCTCTGTGTAAAAAGCA
AH3.05 TacT3 F SalI	GATCGTCGACGGACATGAAGAAATGATGTTTACAG
AH3.06 TacT3 R HindIII	GATCAAGCTTGCCTCATGTTGTCATAACCTACC
AH3.65 GmvT F SalI	GATCGTCGACTGGCGGTAAAGCCTGAATGGAAATAAATGTCACCGCG
AH3.66 GmvT R HindIII	GATCAAGCTTTCAGACTTTATAAAACAAGGTATTAG
AH3.67b KacT F SalI	GATCGTCGACAGGTAAGGTAGAGCTAATGGAACAGCAGCTGACC
AH3.68b KacT R HindIII	GATCAAGCTTCTAGCTTTCATCATCGGTAAACAGC
AH4.35c TacTSce F EcoRI	GATCGAATTCTTAAAGAGGAGAAATTAACTGTGAAAAGCAATACTGATT CGAAG
AH4.36 TacTSce R SalI	GATCGTCGACTTAACACTGGGTGTTTTGATCC
AH4.22 TacTRet F SalI	GATCGTCGACTTAAAGAGGAGAAATTAACTATGAAATTTGAACTGCTGG ATCC
AH4.23 TacTRet R HindIII	GATCAAGCTTCTAATCCAGCAGGCGATCC
AH4.26 TacTBcn F SalI	GATCGTCGACTTAAAGAGGAGAAATTAACTATGAGCGGTGCGCAGTTG G
AH4.27 TacTBcn R HindIII	GATCAAGCTTCTACTTCACCGTTGCCAATGGC
3XFLAG_For	AGCTTGACTACAAAGACCATGACGGTGATTATAAAGATCATGACATCG ATTACAAGGATGACGATGACAAGTAACTCGAGT
3xFLAG_Rev	AGCTACTCGAGTTACTTGTCATCGTCATCCTTGTAATCGATGTCATGAT CTTTATAATCACCGTCATGGTCTTTGTAGTCA
rbsVcaT_FSacI	TATTAGAGCTCAAGGAGGATGTACAAATGGCTTTTAAGCCTTGGGCA
rbsVcaA_FSacI	TATTAGAGCTCAAGGAGGATGTACAAATGGCAAAACAAAATGCTTATGC
VcaT_RSalI	TTTAAGTCGACGGAACATGAGCCGTTACGTT

Supplementary Table 3 | Bacterial Strains

Strain Name	Source/Reference
Salmonella enterica subspecies *enterica* serovar Typhimurium str. 12028 Δ*tacAT1*Δ*tacAT2*Δ*tacAT3*	Rycroft *et al*., Nat Commun. 2018; 9:1993
E. coli DH5α	Thermo Fisher Scientific, USA
E. coli BW25113	Baba *et al*., Mol Syst Biol. 2006; 2:2006.0008

Supplementary Table 4 | GNAT Toxins

GNAT Toxin	Sequence	Genome Accession Number	Protein ID
TacT1	GTGGGACGTGTAACAGCACCAGAACCTTTGTCCGCTTTTC<br>ATCAGGTAGCTGAGTTCGTCAGCGGTGAAGCTGTGCTCGA<br>TGACTGGTTGAAGCAAAAGGGCCTCAAAAACCAGGCTCTC<br>GGAGCGGCCAGAACATTTGTGGTGTGCAAGAAAGACACG<br>AAGCAAGTAGCCGGTTTTTACTCTCTGGCCACCGGTAGCG<br>TCAACCATACAGAAGCGACAGGCAACCTTCGGCGTAACAT<br>GCCAGATCCCATCCCTGTCATTATACTTGCCCGTCTTGCT<br>GTCGATCTCTCATTCCATGGAAAAGGGCTTGGTGCTGATT<br>TACTTCATGATGCAGTGCTTCGTTGCTATCGGGTTGCCGA<br>GAATATTGGTGTACGTGCAATCATGGTTCATGCACTTACC<br>GAAGAAGCCAAAAATTTCTACATTCACCATGGTTTCAAATC<br>ATCACAAACTCAGCAGCGAACATTGTTCCTTAGGCTCCCT<br>CAATAG<br><br>MGRVTAPEPLSAFHQVAEFVSGEAVLDDWLKQKGLKNQALG<br>AARTFVVCKKDTKQVAGFYSLATGSVNHTEATGNLRRNMPD<br>PIPVIILARLAVDLSFHGKGLGADLLHDAVLRCYRVAENIGVRA<br>IMVHALTEEAKNFYIHHGFKSSQTQQRTLFLRLPQ*	NC_01685<br>6	WP_00053<br>3909.1
TacT2	ATGATCTCCACCCCTGAGCCGCTTCATGCCGGACATATTC<br>TTACTCCGTTTTGCTGCGGTGTGGATTCTATAGATAACTGG<br>CTGAAACAGCGGGCGATGAAAAATCAGACCACTGGCGCG<br>TCCCGTACCTTTGTGTGTTGTGGCAGCGATTCGAACGTAC<br>TGGCCTATTACTCGCTGGCGTCCAGCGCGGTCACGACGA<br>ATACCTCCCCCGGTCGCTTTCGTCGCAATATGCCTGACCC<br>GATTCCGGTTGTGGTATTGGGGCGTCTGGCGGTGGATAA<br>ATCGCTTCATGGGCAGGGCGTCGCTCGGGCGCTGGTACG<br>CGACGCAGGGCTGCGGGTTATTCAGGTGGCGGAGACTAT<br>CGGCATTCGTGGGATGCTGGTTCACGCCCTGTCGGATGA<br>AGCGCGGGAATTTTATCAGCGGGTGGGGTTTGTACCGTC<br>GCCGATGGATCCGATGATGTTGATGGTGACGTTGGGGGA<br>TTTGGTGGAGAGTGTTTAA<br><br>MISTPEPLHAGHILTPFCCGVDSIDNWLKQRAMKNQTTGASR<br>TFVCCGSDSNVLAYYSLASSAVTTNTSPGRFRRNMPDPIPVV<br>VLGRLAVDKSLHGQGVARALVRDAGLRVIQVAETIGIRGMLV<br>HALSDEAREFYQRVGFVPSPMDPMMLMVTLGDLVESV*	AM933172	WP_00062<br>6100.1
TacT3	ATGATGTTTACAGACTGGCATGAGGCCGCGATAGGGAAAA<br>CCCACAATCGAATGAATTTTGATTGTGGGGATGCCGATCT<br>GAACCAATTCCTGCAACGCCATGCACGACAAAATCATGAG<br>AAAGGGACAACGAAAACCTATGTTGCGCTTGATAATTCGG<br>ATGTTACACGTATCCACGGCTTTTACTCAGTCAGTCCTGCA<br>TCACTGATATATGCACAGGTTCCCGGCGCAATCAGCAAAG<br>GATTAGGGAGATACGATGTGCCGGTGTTTCGTCTGGGTCG<br>TTTAGCCGTAGACAAATCTATGCAGGGGCAAGGGCTGGG<br>AGCACAACTTTTGTTATCTGCCGGAAAGCGTTGCATACAG<br>GCGGCTTTGCAGGTCGGTGGCGTAGCCCTACTTATTGATG<br>CCAAAAATAAACAGGTCTGCGACTGGTACAAAGGATTTGG<br>CGCAGTACCATTAAACGATCAACCCCTTTCCTTGTTGCTGT<br>CGTTTAAAACGCTTTATGCTGCTTTATCTGCATCTGGTAGG<br>TTATGA	NC_01685<br>6	WP_00097<br>1655.1

	MMFTDWHEAAIGKTHNRMNFDCGDADLNQFLQRHARQNHE KGTTKTYVALDNSDVTRIHGFYSVSPASLIYAQVPGAISKGLG RYDVPVFRLGRLAVDKSMQGQGLGAQLLLSAGKRCIQAALQ VGGVALLIDAKNKQVCDWYKGFGAVPLNDQPLSLLLSFKTLY AALSASGRL*		
ItaT	ATGGTCGACAAGCATGAAGAGATTACTCTGCCCATAGTCC TCTCCTGTAATTATCAGTCTGATATTACTTATCCTGGGCAA AAACAGTTTGATTGCGGTAACCCTGTTATCGATAAATTTGT ACGCGCATCGCTAAAGAAAAGTGTGCGTAATAGCGACTGT GCGGCTAAAGCACTTATTGACAGACAAAGTGGTGAACTGA TCGGCATCTGTACTTTTACGGCATATTCGCTGGAAAAACAA CGCGTTTCTGGCGTCCTTCAGGGTTCACAACCTTCAGAAA TTGGTGTTGTCAGATTAGTCATGTTGGGGGTAGCACGGAA GTATCAAAAGCGGGGCTTTGGTCAGGACCTACTATGTGAT TTTTTTGAACATGTAAAAATAATTCACCAGGCATTACCAATT AAAGGGGTTTATCTTGATGCTGACCCTGCCGCCATTAATTT TTATGCTCGTCTCGGCTTTGTTCAGCTTTCAGCGACACCAA ATGCTTTTGGTGCTGTACCTATGTTTTTGGCGATTCAGCAT ATTCTCGCGGCTTAG<br><br>MVDKHEEITLPIVLSCNYQSDITYPGQKQFDCGNPVIDKFVRA SLKKSVRNSDCAAKALIDRQSGELIGICTFTAYSLEKQRVSGV LQGSQPSEIGVVRLVMLGVARKYQKRGFGQDLLCDFFEHVKI IHQALPIKGVYLDADPAAINFYARLGFVQLSATPNAFGAVPMF LAIQHILAA*	NC_009800	WP_000227784.1
*AtaT	ATGGATGATCTGACGATAGAGATTCTGACCGATGATGCAG ATTATGATCTACAGCGATTCGACTGCGGCGAGGAAGCGTT AAATCTCTTTCTGACGACACATCTCGTTCGTCAACATCGCA ACAAAATTCTGCGAGCGTATATCCTTTGTCGCAACACTCCA GAACGTCAGGTGCTGGGATATTACACATTATGCGGCAGTT GTTTTGAACGAGCCGCATTGCCCTCGAAATCGAAACAGAA AAAAATTCCCTACAAAAATATTCCCAGCGTTACTCTTGGGC GTCTGGCAATTGATCGTTCATTACAGGGGCAGGGATGGG GAGCAACACTGGTTGCTCATGCCATGAACGTCGTCTGGTC AGCCTCTTTAGCGGTAGGTATTCACGGTCTTTTTGTCGAG GCGCTGAATGAAAAAGCCCATACGTTTTATAAATCGCTGG GCTTTATCCCTTTAGTCGGGGAAAACGAAAATGCGTTATTT TTCCCAACCAAATCCATTGAACTGCTTTTTACACAGAGCGA TTAA<br><br>MDDLTIEILTDDADYDLQRFDCGEEALNLFLTTHLVRQHRNKI LRAYILCRNTPERQVLGYYTLCGSCFERAALPSKSKQKKIPYK NIPSVTLGRLAIDRSLQGQGWGATLVAHAMNVVWSASLAVGI HGLFVEALNEKAHTFYKSLGFIPLVGENENALFFPTKSIELLFT QSD*	NC_002655	WP_000342450.1
*KacT	ATGGAACAGCAGCTGACCATTGAAATGATTGCGGATGCGT TTAGCTATGATATTACCGGCTTTGATTGCGGCGAAGAAGC GCTGAACACCTTTCTGAAAGAACATCTGAAACGCCAGCAT GATGGCCAGATTCTGCGCGGCTATGCGCTGGTGAGCGGC GATACCGTGCCGCGCCTGCTGGGCTATTATACCCTGAGC GGCAGCTGCTTTGAACGCGGCATGCTGCCGAGCAAAACC CAGCAGAAAAAAATTCCGTATCAGAACGCGCCGAGCGTGA CCCTGGGCCGCCTGGCGATTGATAAAAGCGTGCAGGGCC AGGGCTGGGGCGAAATGCTGGTGGCGCATGTGATGCGCG TGGTGTGGGGCGCGAGCAAAGCGGTGGGCATTTATGGCC TGTTTGTGGAAGCGCTGAACGAAAAAGCGAAAGCGTTTTA	NC_016845	WP_002887280.1

	TCTGCGCCTGGGCTTTATTCAGCTGGTGGATGAAAACAGC<br>AACCTGCTGTTTTATCCGACCAAAAGCATTGAACAGCTGTT<br>TACCGATGATGAAAGCTAG<br><br>MEQQLTIEMIADAFSYDITGFDCGEEALNTFLKEHLKRQHDG<br>QILRGYALVSGDTVPRLLGYYTLSGSCFERGMLPSKTQQKKI<br>PYQNAPSVTLGRLAIDKSVQGQGWGEMLVAHVMRVVWGAS<br>KAVGIYGLFVEALNEKAKAFYLRLGFIQLVDENSNLLFYPTKSI<br>EQLFTDDES*		
GmvT	ATGGAAATAAATGTCACCGCGCCAGCATTGTTGACGGATG<br>AGCATATACTTCAGCCATTTGACTGTGGAAATGAGGTGCT<br>AAGTAACTGGTTACGTGGTCGGGCTATGAAAAACCAGATG<br>CTCAATGCTTCTCGCACGTTCGTTATTTGCTTGGAGGATAC<br>TTTACGTATTGTGGGATACTACTCATTAGCTACTGGTTCCG<br>TCACTCATGCCGAGCTTGGTCGCAGTTTGCGGCATAACAT<br>GCCCAATCCTGTCCCTGTTGTTCTGTTAGGGCGTCTGGCT<br>GTTGATGTTTGTACCCAGGGGCATGGTTTCGGTAAATGGC<br>TACTAAGTGATGCCATTCACCGGGTTGTCAATCTGGCTGA<br>TCAGGTTGGCATTAAGGCTGTTATGGTACATGCAATTGAT<br>GATGACGCCAGAGCATTTTATGAGCGTTTTGGTTTTGTTCA<br>GTCAGTTGTGGCACCTAATACCTTGTTTTATAAAGTCTGA<br><br>MEINVTAPALLTDEHILQPFDCGNEVLSNWLRGRAMKNQML<br>NASRTFVICLEDTLRIVGYYSLATGSVTHAELGRSLRHNMPN<br>PVPVVLLGRLAVDVCTQGHGFGKWLLSDAIHRVVNLADQVGI<br>KAVMVHAIDDDARAFYERFGFVQSVVAPNTLFYKV*	AF348706	WP_00040<br>5245.1
$TacT_{Bcn}$	ATGAGCGGTGCGCAGTTGGTAGTGGCCGCCCTTGAGGCG<br>GCTCATGATCGATCTCGATTCGCATGCGGCACGCCCGCG<br>CTTGATCGATATCTGCGCGAAGTGGTTACGCAGGACGTAC<br>GCCGCAGGGTGGCCGCCTGTTTCGTGATGCTCGACGGCA<br>ACGTCGTCGCGGGCTACTACACACTGTCTGCCGCCAGCG<br>TCGCGCTCACGGATTTGCCGCACGCCATCGCGCGCAAGT<br>TGCCCCGTTATCCTGCGATCCCGGTGGTGCGCATGGGGC<br>GCCTGGCCGTCGATCAGGCATATCGCGGCAGGCGACTCG<br>GCGCAGCGCTGCTGGTCAACGCGCTGCAACGTGCCGCAA<br>AGTCCGAAATCGCTGCGGTGGCTCTGACGGTTGACGCAA<br>AGGACGAAACGGCGGCGGCGTTCTACCGGCATTTCGGCT<br>TCGCGCCGCTGACTAGCGACCCGCTGGCATTGTTCATGC<br>CATTGGCAACGGTGAAGTAG<br><br>MEINVTAPALLTDEHILQPFDCGNEVLSNWLRGRAMKNQML<br>NASRTFVICLEDTLRIVGYYSLATGSVTHAELGRSLRHNMPN<br>PVPVVLLGRLAVDVCTQGHGFGKWLLSDAIHRVVNLADQVGI<br>KAVMVHAIDDDARAFYERFGFVQSVVAPNTLFYKV*	AM747721	WP_00040<br>5245.1
AtaT2	ATGGGAATAACGGCTCCTACTCCCCTAACGTCTGAACATA<br>ATCTGGCTGACTTTTGCTGTTCCGATCACGGGATGAATGA<br>ATGGCTAAAAAAGAAAGCGTTAAAAAATCACAGCTCCGGT<br>CTGTCTCGTGTTTACGTTATCTGTATTGCCAATACCCGCCA<br>GGTAATCGGCTATTACTGCCTTTCTACAGGCAGTATTCAAC<br>GTAATTTGGCCCCTGGAGCCATGCGACGTAATGCTCCCGA<br>ATCGTTGCCTGTTGTCGTATTAGGCAGACTGGCAATTGAT<br>CAAGCCTGGGCAGGTAAAGGGTTGGGCGTCGCATTGTTG<br>AAAGATGCAGTCTATCGCACAATGTCCATAGCTCAACAGG<br>TAGGGGTTCGTGCGCTCATTGTTCATGCCTTAGACGATTC<br>AGTACGCAATTTCTATCTGAAGTATGCCTTTGTACCTTCAC	NC_00269<br>5	WP_00130<br>1452.1

	CATTCCAGTCACTTACCTTACTGTATCCCATTACTCTGGAG<br>TAA<br><br>MGITAPTPLTSEHNLADFCCSDHGMNEWLKKKALKNHSSGL<br>SRVYVICIANTRQVIGYYCLSTGSIQRNLAPGAMRRNAPESLP<br>VVVLGRLAIDQAWAGKGLGVALLKDAVYRTMSIAQQVGVRAL<br>IVHALDDSVRNFYLKYAFVPSPFQSLTLLYPITLE*		
*TacT$_{Ret}$	ATGAAATTTGAACTGCTGGATCCGAAACGCCATGATCGCG<br>AAGGCTTTGATTGCGGCGTGGTGGCGCTGAACACCTATCT<br>GCGCCGCTTTGCGAACCAGGATATTAAACGCGGCCTGAC<br>CCGCGTGTATGTGCTGTGCGATGAAGCGCGCATTGCGGG<br>CTATTTTAGCCTGAGCGCGCATAGCGTGAGCCGCCAGGAT<br>CTGCCGCCGAAAATTCAGGCGGGCCCGTATGAAGAACTG<br>CCGTTTCTGATTCTGGGCCGCCTGGCGGTGGATCGCGAA<br>TATCAGGGCCGCGGCCTGGGCGATGCGCTGATTGTGCAT<br>GCGTTTGCGATTACCCGCAGCGCGGCGGGCCAGATTGGC<br>ATTCTGGGCATGATTGTGGATGCGAAAGATGAACGCGCG<br>GCGGATTTTTATCAGCGCTTTGGCTTTCGCCGCCTGAGCG<br>GCGCGGCGCTGCGCCTGGTGCTGCCGATTACCGCGATGG<br>ATCGCCTGCTGGATTAG<br><br>MKFELLDPKRHDREGFDCGVVALNTYLRRFANQDIKRGLTR<br>VYVLCDEARIAGYFSLSAHSVSRQDLPPKIQAGPYEELPFLIL<br>GRLAVDREYQGRGLGDALIVHAFAITRSAAGQIGILGMIVDAK<br>DERAADFYQRFGFRRLSGAALRLVLPITAMDRLLD*	NZ_CP006<br>986	WP_03868<br>9238.1
TacT$_{Sce}$	ATGAAAAGCAATACTGATTCGAAGTTAAAACTAGTATCTGG<br>TGATTCTGATATTCAGTTTAGCGGGATCAAGAAGTTTGATT<br>GTGGCGACAGAGTGCTGAACAAATTTCTGGAACAGCTAAA<br>GCGCCAGTGTAGCCGTGACAATATAAAAGCCCTTGTATTG<br>ATTGATGATAGTAATCAGGTGGCAGGATTTGTTACGGCTT<br>CTTTGTATCAGTTAGGGAAGGAACGTATCCCTGATGACAC<br>GTTCCCATACGCACCTCCTCCTTTAGTTGCTGTAATGAAAA<br>TCCCCATGATTGCCGTCGATAAAGAATACCAAAGGCAGGG<br>CTGGGGAGTTCAGTTGATGCGCGCTGTTCTTGATTATGCG<br>CTTGAAAGTGCGGAACAGGTAAAAGGTATAAAAGGTGTCT<br>ATCTTGATGCCAAAGTTGATGCCCGAAGCTTCTATGAGGA<br>CCTGGGCTTTGACGCGATAAGTGAGGATGTTAGTCTAAAT<br>GGTACTGTACCGATGTTCATTTCGATGGATACGTTACGTG<br>ACAGTAAAATGGATCAAAACACCCAGTGTTAA<br><br>MKSNTDSKLKLVSGDSDIQFSGIKKFDCGDRVLNKFLEQLKR<br>QCSRDNIKALVLIDDSNQVAGFVTASLYQLGKERIPDDTFPYA<br>PPPLVAVMKIPMIAVDKEYQRQGWGVQLMRAVLDYALESAE<br>QVKGIKGVYLDAKVDARSFYEDLGFDAISEDVSLNGTVPMFIS<br>MDTLRDSKMDQNTQC*	NZ_CP012<br>835	WP_02323<br>3612.1
*AcaT	ATGGCGACTGAGGAAACAGAGAATCCGAAGATCGAACGT<br>CTGACAGCGGAACACTTAAGTATGGAGTTTAATTCATCGA<br>GTGAACGCATGAACCGCTTCTTTGCGGAGTTCGCCTTACA<br>AGAGCAAGCATTGGGTATTTCATCTACTTACTGCTTGGTAG<br>ATGAAGATGGGGTATTAGGGTTTTTCACGCTGTGCCAGGG<br>ATCTGTGTTAAAAGAGGTGTTGAAGTACAAGACTCCATATA<br>AGGACGTTCCAGTCTTCCGCATTGGACGTTTAGCGGTGAC<br>TGAAGAGTGCGAGCGCACAGGAATCGGACGCGCATTGGT<br>TGCGATTGCTTTcAAgAAAGCGTCCGAAGCCCGTATTACTG<br>CGGGCGCGGTTGCGCTGGTCGTCGATGCTGTTCCTAGTG<br>CAGTAGATTTTTATCGaAAATGCAACTTTGAGGCCGTTCAA	NZ_AP024<br>137	WP_20221<br>1968.1

	GAGAGTCCGGGTTCTAAAACTGTATTCATGGCGCTGTCAC TTGGGGATAAGGAGGAGTAA<br><br>MATEETENPKIERLTAEHLSMEFNSSSERMNRFFAEFALQEQ ALGISSTYCLVDEDGVLGFFTLCQGSVLKEVLKYKTPYKDVP VFRIGRLAVTEECERTGIGRALVAIAFKKASEARITAGAVALVV DAVPSAVDFYRKCNFEAVQESPGSKTVFMALSLGDKEE*		
*SonT	ATGTCTACGAACTACATTGACTGTCAATTGCTGAACAAAAT GGAGCGCCAGCCTAGTTTTTCGTCGTTTGACTGCGGTGAT CCTTTCTTGGATTCCTTCGCTCCCAAGAAGTTAGCGAACG CAGACGCAAATAACGATTCTCGTGTTTACGTTGCTGTAGAT GGAGACATTGGTGTCGGATATGCGACAATGAAAGTGTTCA TGTTGAGCAACGACGAATACAAGATTTTGAGTGGAAAGTA CCCTCGTCAGGTGCCAGTAGTCATGTTAGATCAAATCGCA GTAGACAAGGCATACCAGGGTAAGGGCATTGGTAAGCGTT TAATGCGTAAGGTATTGGAGGCTACTGTTTTGGTAAATGAA TTAGCTGCCGCTAAGGGGTTGGCACTGTGGGCTCACCCA CGTGCTAAAGACTTCTATGAAAGTCTGGGATTTGAAGCTAT CCCAGATGCTACCAAGCAGGTCCAAGACGTAGAGTTAACA CTTATGTTTATCCATGTGGAAACGATTTTAGACGCTTTGAA GTAA<br><br>MSTNYIDCQLLNKMERQPSFSSFDCGDPFLDSFAPKKLANA DANNDSRVYVAVDGDIGVGYATMKVFMLSNDEYKILSGKYP RQVPVVMLDQIAVDKAYQGKGIGKRLMRKVLEATVLVNELAA AKGLALWAHPRAKDFYESLGFEAIPDATKQVQDVELTLMFIH VETILDALK*	NC_004349	WP_011074389.1
*VcaT	ATGGCTTTTAAGCCTTGGGCAACCGTCGAAAAAGTTGGCG GGCAAAACCTGTCTAACTTTACGTGCGGAGTAGGTAATTT CGACTCCTGGCTTAAAAATGAAGCAGAAAGTCAGCAAAGA CAAGGCAGAGTCACAACTTGGCTAGCAGTAGATAGGGATG GAGAAGTTGTAGGGTATTTTTCGCTAAGACACGCCGTTTTT GTCGCTGGAAACCTCCAGCTAAGTAAGACCCGAGCGAAG AAACTCGGATTAGAAGACGGGGCGAGTTCAGGGATTCTGA TAGCAAAACTCGCTTTACATAAAAAATGGCAAGGAAAAGG ATGTGGCCTGCTGCTACTGGACGAAGCCCTAGCAAAATGT GTCCAAGTTTTTAATACAGCCGCCTACCAACTGATAATGGT GGACGCAGCAAGACCCGAATTGGTTTTGTTTTACGAACAA AGAGGCTTCTTCCCGAGCGGGGAAAACCTGCGACTGATA AACACTGTTAGATCGATAGCTTCCGATAAAAGAAACGGCT CATGTTCCTAA<br><br>MAFKPWATVEKVGGQNLSNFTCGVGNFDSWLKNEAESQQR QGRVTTWLAVDRDGEVVGYFSLRHAVFVAGNLQLSKTRAKK LGLEDGASSGILIAKLALHKKWQGKGCGLLLLDEALAKCVQV FNTAAYQLIMVDAARPELVLFYEQRGFFPSGENLRLINTVRSI ASDKRNGSCS*	NZ_PKHX01000000	WP_101930441.1

*Sequence codon optimized for *E. coli* expression
